# Overexpression of miR-124 in Motor Neurons Plays a Key Role in ALS Pathological Processes

**DOI:** 10.3390/ijms22116128

**Published:** 2021-06-07

**Authors:** Ana Rita Vaz, Daniela Vizinha, Hermes Morais, Ana Rita Colaço, Gecioni Loch-Neckel, Marta Barbosa, Dora Brites

**Affiliations:** 1 Neuroinflammation, Signaling and Neuroregeneration Group, Research Institute for Medicines (iMed.ULisboa), Faculty of Pharmacy, Universidade de Lisboa, 1649-003 Lisbon, Portugal; daniela.vizinha@gmail.com (D.V.); h.morais@campus.fct.unl.pt (H.M.); anarac97@gmail.com (A.R.C.); gneckel@hotmail.com (G.L.-N.); mbarbosa@ff.ulisboa.pt (M.B.); 2 Department of Pharmaceutical Sciences and Medicines, Faculty of Pharmacy, Universidade de Lisboa, 1649-003 Lisbon, Portugal

**Keywords:** amyotrophic lateral sclerosis, inflamma-miRNAs, miR-124 modulation, motor neuron regeneration, motor neuron secretome, mSOD1 mice model, neuro-immune deregulation, paracrine signaling regulation, spinal microglia, spinal organotypic cultures

## Abstract

miRNA(miR)-124 is an important regulator of neurogenesis, but its upregulation in SOD1G93A motor neurons (mSOD1 MNs) was shown to associate with neurodegeneration and microglia activation. We used pre-miR-124 in wild-type (WT) MNs and anti-miR-124 in mSOD1 MNs to characterize the miR-124 pathological role. miR-124 overexpression in WT MNs produced a miRNA profile like that of mSOD1 MNs (high miR-125b; low miR-146a and miR-21), and similarly led to early apoptosis. Alterations in mSOD1 MNs were abrogated with anti-miR-124 and changes in their miRNAs mostly recapitulated by their secretome. Normalization of miR-124 levels in mSOD1 MNs prevented the dysregulation of neurite network, mitochondria dynamics, axonal transport, and synaptic signaling. Same alterations were observed in WT MNs after pre-miR-124 transfection. Secretome from mSOD1 MNs triggered spinal microglia activation, which was unno-ticed with that from anti-miR-124-modulated cells. Secretome from such modulated MNs, when added to SC organotypic cultures from mSOD1 mice in the early symptomatic stage, also coun-teracted the pathology associated to GFAP decrease, PSD-95 and CX3CL1-CX3CR1 signaling im-pairment, neuro-immune homeostatic imbalance, and enhanced miR-124 expression levels. Data suggest that miR-124 is implicated in MN degeneration and paracrine-mediated pathogenicity. We propose miR-124 as a new therapeutic target and a promising ALS biomarker in patient sub-populations.

## 1. Introduction

Amyotrophic lateral sclerosis (ALS) is a neurodegenerative disease that affects motor neurons (MNs), though it is recognized that glial cells, namely astrocytes and microglia, are also involved in ALS pathogenesis and progression [1,2]. Mice carrying mutations in superoxide dismutase 1 (mSOD1) are the most used models to investigate ALS pathophysiology. They closely mimic ALS symptoms in humans, and distinct glial phenotypes and inflammatory-associated profiles were identified in these animals [3,4,5].

Neuronal degeneration in ALS is linked to mitochondrial dysfunction, endoplasmic reticulum stress, unfolded protein response malfunction, and autophagy impairment [6,7,8]. However, the molecular mechanisms underlying MN-glia deregulated paracrine signaling in ALS are not fully elucidated. In recent years, microRNAs (miRNAs), small noncoding RNAs, were described as active drivers in intercellular communication, as they affect gene expression in both donor and recipient cells [9]. miRNAs are released from cells into their secretome and circulate either as soluble molecules or as cargo in small extracellular vesicles with ~100 nm size, also called exosomes. Once collected by the target cell, extracellular miRNAs regulate RNA availability and protein translation. These properties turn miRNAs good targets for therapeutic intervention, since their modulation can simultaneously have benefits over the cells of origin and the neighboring ones. Although dysregulated miRNA profiles were found in ALS microglia [10], and in sporadic ALS (sALS) patients [11], the role of secretome containing specific inflammatory-associated miRNAs (inflamma-miRNAs) in the disease is not known. Several miRNA alterations are reported in the blood, cerebrospinal fluid (CSF), and post-mortem tissue, either in the brain or in the spinal cord (SC) of ALS patients, highlighting their potential implication in ALS pathology [12,13]. miR-124 is the most abundant in the CNS and its expression is crucial for neuronal function, namely for synaptic plasticity, regulation of neurite outgrowth, and neuronal/astrocyte differentiation [14,15]. miR-124 is also considered to be a negative regulator of inflammation, namely in microglial cells [16,17]. In mSOD1 transgenic mice, miR-124 transported in MN-derived exosomes was shown to upregulate the expression of glutamate transporter-1 (GLT-1) in astrocytes, thus suggesting a protective role [18]. However, increased levels of miR-124 were reported in SC and brainstem of mSOD1 mice at symptomatic stages, and found to be differently expressed in some locus of neurodegeneration [19]. In the same study, miR-124 was downregulated in neural stem cells from mSOD1 mice, but upregulated in differentiated MNs, suggesting its involvement in cellular differentiation. miR-124 overexpression also revealed to have a negative impact on MN morphology and mitochondrial activity, where vimentin was identified as a direct target [20]. These authors suggested that miR-124 expression should then be kept within defined limits.

Upregulation of miR-124 and miR-125b was found in the brain of ALS mice at late disease stages, where the first was associated with neural activity and the later with corticospinal tract degeneration [21]. Their elevation in the CSF of ALS patients was linked to neuronal demise and neuroinflammation/gliosis, respectively, as well as with disease severity, suggesting miR-124 upregulation as a potential biomarker [22,23,24]. Our studies in the SC of mSOD1 mice demonstrated that miR-124 and miR-146a only increased in the symptomatic phase, while miR-155 upregulation was observed in early stages [3], reinforcing the relevance of neuroinflammation in the disease. Moreover, elevation of miR-124 not only showed to rise in MN-like cells overexpressing mSOD1, but also to be carried in their derived exosomes, that once internalized by microglia affect their immune function with acute and delayed effects [25].

However, it is not clear how neuronal vitality is affected by miR-124 expression levels in ALS MNs, how it impairs the function of their neighboring glial cells, and whether its modulation toward stipulated expression values can be considered as an effective therapeutic strategy in the reestablishment of cellular homeostasis.

In this study, we used the mouse MN-like cell line NSC-34 overexpressing human SOD1, either wild type (WT) or mutated in G93A (mSOD1) [26], with the following objectives: (i) to assess if the upregulation of miR-124 in WT MNs was translated into dysfunctionalities comparable to those of mSOD1 MNs; (ii) to determine whether the downregulation of miR-124 in mSOD1 MNs was effective in recovering cell function; (iii) to explore if the secretome from mSOD1 MNs induced microglia polarization, but not the one from anti-miR-124-treated cell; and (iv) to investigate alterations of neuronal glial homeostasis in the SC of mSOD1 mice after disease onset, their exacerbation by the addition of secretome from mSOD1 MNs, and potential recovery by that from anti-miR-124-treated mSOD1 MNs. For that, WT MNs were transfected with pre-miR-124 and mSOD1 MNs with anti-miR-124, to upregulate or to restore miR-124 normal levels, respectively. Neuronal synaptic and mitochondria dynamics were evaluated, as well as inflammatory miRNAs in cells and their secretome. To assess the effects of the secretomes from the non-treated and anti-miR-124-treated mSOD1 MNs on microglia polarization, microglial cells were isolated from the SC of WT mice pups and maintained for 2 days in vitro (2DIV) after being 21 DIV in mixed glial culture [27]. To further evaluate the harmful (the first) and beneficial (the second) effects of such secretomes, we used SC organotypic cultures from mSOD1 mice at early symptomatic stage (10–12 weeks-old), which are considered the best way to preserve cell–cell interactions and the biochemical organization of cells in the SC [28,29].

Data reveal that upregulation of miR-124 is associated with mSOD1 MN degeneration, deregulated neuro-immune crosstalk, and homeostatic imbalance, supporting its potential as a disease biomarker. Our findings further indicate that targeting and restoring normal neuronal miR-124 levels in ALS may hold promise for treating people with the disease.

## 2. Results

### 2.1. Overexpression of miR-124 in mSOD1 MNs and in Pre-miR-124-Treated WT MNs Compromise Cell Viability, Which Is Prevented by Restoring Its Normal Values

Despite the critical role of miR-124 in neuronal differentiation and neurite outgrowth [30], its increased levels were associated with pathological events in neurodegenerative diseases, including ALS [24,31]. We have previously demonstrated that miR-124 is upregulated in NSC-34 mSOD1 MNs [25]. These cells were used in the present work to decipher the mechanisms underlying MN degeneration in ALS disease, once they were well characterized in several studies [32,33], including our ones [4,25,26].

**Figure 1:**
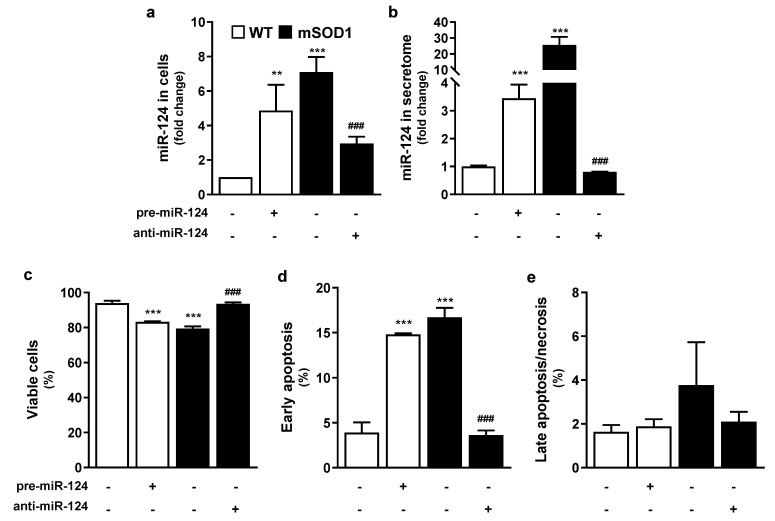
Upregulation of miR-124 in WT MNs increases cell death by apoptosis as occurs in mSOD1 MNs, which survival is restored by treatment with anti-miR-124. After 2 days of differentiation, NSC-34 WT and mSOD1 MNs were transfected with pre-miR-124 or anti-miR-124, respectively, for 12 h and maintained for additional 48 h. (**a**,**b**) Cells and derived secretome were collected for miRNA isolation and levels of miRNA-124 were evaluated by reverse transcriptase quantitative real-time PCR (RT-qPCR). SNORD110 and RN1UA1 were used as reference genes. (**c**–**e**) Percentage of viable, early apoptotic, and late-apoptotic/necrotic cells were determined by flow cytometry with phycoerythrin-conjugated annexin V (annexinV-PE) and 7-amino-actinomycin D (7-AAD). The three populations were distinguished as follows: (**c**) viable cells (annexin V-PE and 7-AAD negative); (**d**) early apoptotic cells (annexinV-PE positive and 7-AAD negative); and (**e**) cells in late stages of apoptosis or necrosis (annexinV-PE and 7-AAD positive). Results are mean ± SEM from at least six independent experiments and expressed as fold change vs. WT MNs (**a**,**b**), or as percentage per total number of counted events (**c**–**e**). *** *p* < 0.001 and ** *p* < 0.01 vs. WT MNs; ^###^ *p* < 0.001 vs. mSOD1 MNs; one-way ANOVA followed by multiple comparisons Bonferroni post-hoc correction. MNs, motor neurons; mSOD1, MNs overexpressing G93A mutation in superoxide dismutase 1; WT, wild type.

Our first aim was to explore if miR-124 overexpression was directly associated with NSC-34 mSOD1 MN reduced survival [26]. For that, we transfected WT MNs with pre-miR-124 for 12 h and cells were analyzed 48 h after transfection. As indicated in Figure 1a, treatment of WT cells with pre-miR-124 led to ~5-fold increased expression (*p* < 0.01 vs. untreated WT cells), only slightly below that in mSOD1 MN (~8-fold increase, *p* < 0.001 vs. untreated WT cells), thus confirming transfection efficiency. In the same way, pre-miR-124 in WT cells induced the release of increased miR-124 levels into the MN secretome (*p* < 0.001 vs. that of untreated WT cells), as depicted in Figure 1b, recapitulating what occurred with the secretome from mSOD1 MNs (*p* < 0.001 vs. that of untreated WT cells). To note, however, that the enrichment was near 4.5-fold in the WT MNs treated with pre-miR-124, but the original one from mSOD1 MNs was around 25.7-fold, as compared to control values. This finding suggests that miR-124, besides being actively produced by mSOD1 MNs, is cleared from the cells into the extracellular milieu with expected consequences in homeostatic imbalance.

We then decided to transfect mSOD1 MNs with anti-miR-124 (Figure 1a,b). As anticipated, such modulation was effective in switching miR-124 levels close to control ones, either in cells (partially), or in their secretome (totally) (*p* < 0.001 vs. untreated WT MNs). Results from mock controls (Optimem plus transfection reagent), and respective Pre-miR Negative Control (in WT MNs) or Anti-miR Negative Control (in mSOD1 MNs) were like the non-treated ones, i.e., WT or mSOD1 MNs incubated with Optimem for 12 h, validating the miR-124 specificity (Figure S1).

Having optimized the upregulation of miR-124 in WT cells and its downregulation in mSOD1 MNs, we then assessed the harmful (first case) and the beneficial (second case) effects on cell viability, as well as in cell death by early apoptosis or late apoptosis/necrosis (Figure 1c–e). Pre-miR-124 transfection in WT cells caused a significant reduction in MN viability and enhanced cell death by early apoptosis (*p* < 0.001 for both vs. untreated MNs) similarly to mSOD1 MNs findings (*p* < 0.001 for both vs. untreated MNs) (Figure 1c–e). In contrast, anti-miR-124 treatment of mSOD1 MNs completely abrogated cell demise. No changes were observed in any of the conditions for late apoptosis or necrosis (Figure 1e).

Data indicate that miR-124 expression, if surpassing a certain expression level, may compromise MN vitality, and that its modulation toward control levels may have therapeutic benefits, considering that it simultaneously targets many transcripts and fine tunes complex signaling pathways [9,34,35].

### 2.2. Modulation of miR-124 toward Normal Values in mSOD1 MNs Reverts Their Aberrant Inflamma-miRNA Profile and Partialy Recovers WT Secretome Homeostatic Signature

To investigate whether up- and downregulation of miR-124 in MNs can modify the expression of other inflamma-miRNAs, such as miR-125b, miR-146a, and miR-21, we assessed their expression levels in the established conditions. These miRNAs were recognized to be dysregulated in different tissue sources from ALS patients [12], as well as in the brain and SC of symptomatic mSOD1 mice [3,4].

**Figure 2:**
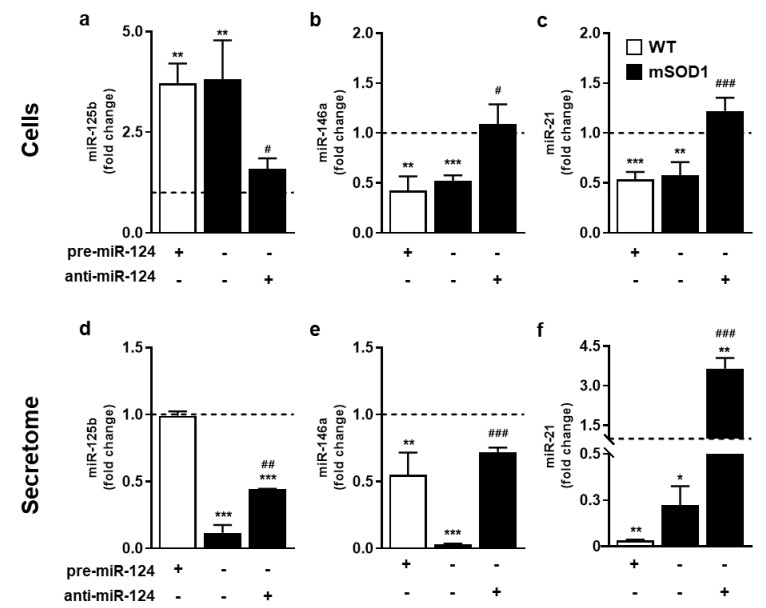
Targeting of miR-124 toward normal values in MNs enriched in miR-124 abrogates miR-125b overexpression and causes miR-146a/miR-21 downregulation in both cells and secretome, thus inhibiting a pathological inflamma-miRNA profile in mSOD1 MNs. After 2 days of differentiation, NSC-34 WT and mSOD1 MNs were transfected with pre-miR-124 or anti-miR-124, respectively, for 12 h and maintained for additional 48 h. Cells and derived secretome were collected for miRNA isolation. SNORD110 and RN1UA1 were used as reference genes. (**a**,**d**) miRNA-125b, (**b**,**e**) miRNA-146a, and (**c**,**f**) miR-21 levels in cells and their derived secretome were evaluated by reverse transcriptase quantitative real-time PCR (RT-qPCR). Results are mean ± SEM from at least 6 independent experiments and expressed as fold change vs. WT MNs (dashed line). *** *p* < 0.001, ** *p* < 0.01, and * *p* < 0.05 vs. WT MNs; ^###^ *p* < 0.001, ^##^ *p* < 0.01, and ^#^ *p* < 0.05 vs. mSOD1 MNs; one-way ANOVA followed by multiple comparisons Bonferroni post-hoc correction. MNs, motor neurons; mSOD1, MNs overexpressing G93A mutation in superoxide dismutase 1; WT, wild type.

We found that miR-125b was overexpressed in mSOD1 MNs, while miR-146a and miR-21 were downregulated (Figure 2a–c, at least *p* < 0.01 vs. untreated WT MNs), supporting a specific pathological inflamma-miRNA signature in our model. Interestingly, the same fingerprint of miRNAs in mSOD1 MNs was recapitulated after miR-124 upregulation in WT MNs (at least *p* < 0.01 vs. untreated WT MNs), attesting the miR-124 relevant role, not only in the cell fate, but also in the expression of other immune-associated miRNAs in ALS MNs. Indeed, miR-125b upregulation, together with miR-146 and miR-21 downregulation, were totally reverted after transfection with anti-miR-124 in mSOD1 MNs (at least *p* < 0.05 vs. untreated mSOD1 MNs).

Interestingly, the miRNA profile in the secretome (Figure 2d,e) recapitulated the miR-146a and miR-21 reduction of mSOD1 MNs that was also reproduced by treatment of WT MNs with pre-miR-124 (at least *p* < 0.05 vs. untreated WT MNs). The depressed levels of miR-125b in the mSOD1 MN secretome (*p* < 0.001 vs. untreated WT MNs) contrasted with its elevated representation in cells. This finding indicates a target-dependent intracellular retention of this miRNA in the pathological MNs, once it was not reproduced in the pre-miR-124-treated MNs. Now considering the secretome from the anti-miR-124 modulation in mSOD1 MNs, some recovery was obtained for miR-125 expression (*p* < 0.01 vs. untreated mSOD1 MNs), and almost completely for miR-146a (*p* < 0.01 vs. untreated mSOD1 MNs) with values close to untreated WT cells. Most importantly, marked elevation of miR-21 expression in the secretome from the anti-miR-124 condition recapitulated the effect produced on mSOD1 MNs (*p* < 0.01 vs. untreated mSOD1 MNs), probably accounting to the reversal of the early apoptosis subsequent to miR-124 elevation [36].

### 2.3. Impairment of Neurite Outgrowth, Axonal Transport, Synaptic Signaling, and Mitochondria Dynamics in miR-124-Enriched MNs Is Prevented when miR-124 Normal Levels Are Reestablished in mSOD1 MNs

**Figure 3:**
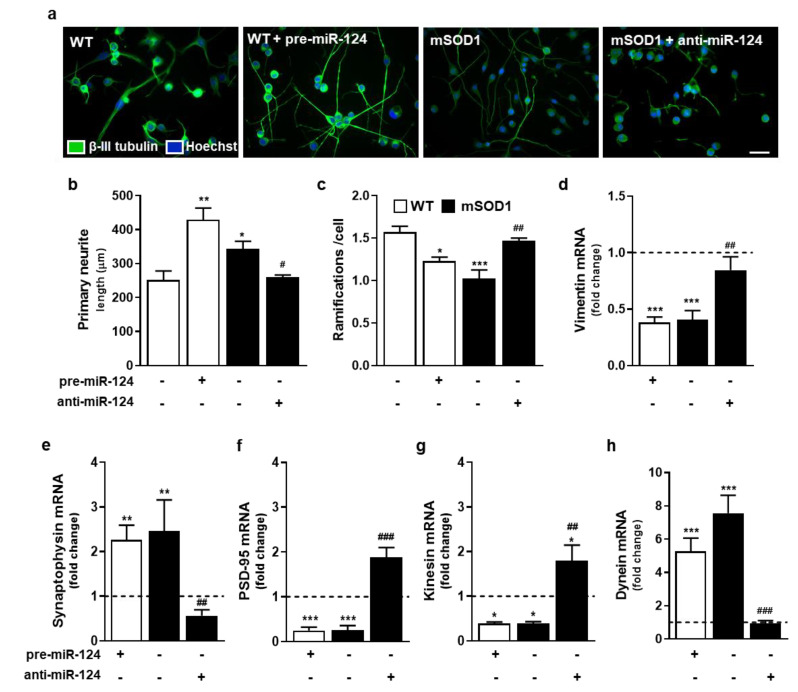
Impaired neuronal arborization, trans-synaptic signaling, and axonal function by elevated levels of miR-124 in MNs is abolished by anti-miR-124 treatment of mSOD1 MNs. After 2 days of differentiation, NSC-34 (wild type, WT) MNs and mSOD1 MNs were transfected with pre-miR-124 or anti-miR-124, respectively, for 12 h and maintained for additional 48 h. After this period, cells were collected for (**a**–**c**) immunostaining analysis or (**d**–**h**) mRNA isolation. MNs were stained with an antibody to β-III tubulin and (**a**) representative images of each condition are shown. (**b**,**c**) Neurite length and ramification number were measured by using NeuroJ (plug-in from ImageJ software). Further, (**d**) vimentin, (**e**) synaptophysin, (**f**) PSD-95 (Dlg4 gene), (**g**) kinesin (Kif5b gene), and (**h**) dynein mRNA levels were evaluated by reverse transcriptase quantitative real-time PCR (RT-qPCR). β-actin was used as the reference gene. Results are mean ± SEM from at least 6 independent experiments and expressed as fold change vs. WT MNs (dashed line). *** *p* < 0.001, ** *p* < 0.01, and * *p* < 0.05 vs. WT MNs; ^###^ *p* < 0.001, ^##^ *p* < 0.01, and ^#^ *p* < 0.05 vs. mSOD1 MNs; one-way ANOVA followed by multiple comparisons Bonferroni post-hoc correction. Scale bar: 20 μm. MNs, motor neurons; mSOD1, MNs overexpressing G93A mutation in superoxide dismutase 1; PSD-95, post-synaptic density protein 95; WT, wild type.

To better understand the consequences of miR-124 differential expression in MN vitality, we next assessed changes in dendritic arborization, as well as in the expression of vimentin, a neurofilament subunit [37] and a direct target of miR-124 [20], together with the perturbation of synaptic and axonal transport dynamics. We observed that both pre-miR-124-treated WT cells and mSOD1 MNs have longer primary neurites, but reduced number of ramifications (at least *p* < 0.05 vs. WT MNs, Figure 3a–c). Increased neurite length and reduced arborization suggest miR-124 upregulation as an adaptive mechanism to compensate deficits in neuron–neuron communication. However, the reduction in branching may compromise the connection with multiple targets. This impairment in neurite network may be associated to the low gene expression of vimentin (Figure 3d) by enhanced miR-124 levels of pre-miR-124-treated WT MNs and mSOD1 cells (*p* < 0.001 vs. untreated WT MNs). This protein, which is known to be associated to neurite outgrowth, was shown to modify its expression levels by damage [38]. Interestingly, treatment with anti-miR-124 in mSOD1 MNs reversed all the effects (at least *p* < 0.05 vs. untreated mSOD1 MNs) and favored neurite branching in this model.

When assessing axonal transport and synaptic dynamics, we observed that compensatory and opposite mechanisms were induced by high or low miR-124 values. Considering synaptic dysfunction, data demonstrated that mSOD1 MNs have increased presynaptic synaptophysin and decreased postsynaptic density protein 95 (PSD-95) gene expression levels (Figure 3e,f; at least *p* < 0.01 vs. untreated WT MNs), confirming trans-synaptic signaling impairment in ALS MNs. These changes were reproduced by upregulation of miR-124 in WT MNs with pre-miR-124 (at least *p* < 0.01 vs. untreated WT MNs). Treatment of mSOD1 MNs with anti-miR-124 completely reversed such alterations and favored connection to the postsynaptic neuron (at least *p* < 0.01 vs. mSOD1 MNs). Levels of miR-124 in MNs also differently influenced the axonal transport. We found that the anterograde transport, mediated by kinesin, was inhibited, while the retrograde one, driven by dynein, was stimulated by increased expression of miR-124 (Figure 3g,h; at least *p* < 0.05 vs. untreated WT MNs), indicating a compromised transmission route. The targeting of mSOD1 MNs with anti-miR-124 empowered the anterograde transport and restored the retrograde normal values, thus favoring synaptic function (at least *p* < 0.01 vs. mSOD1MNs).

**Figure 4:**
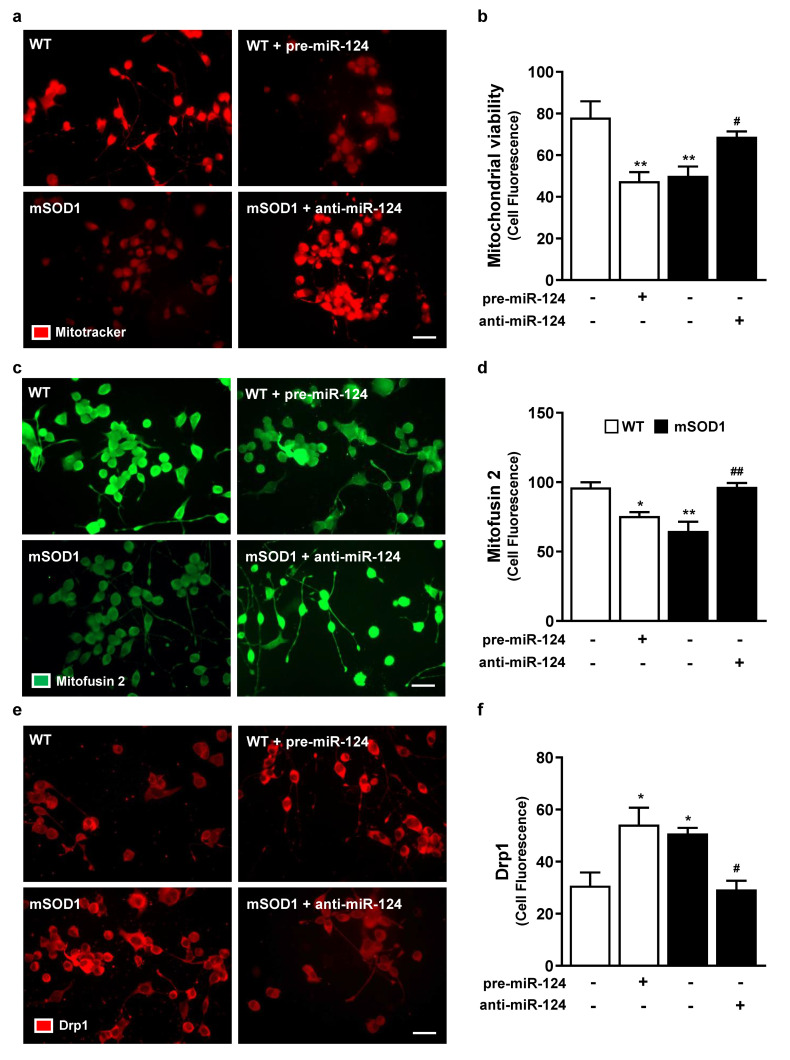
Compromised mitochondrial viability, fusion, and fission in MNs with high levels of miR-124 is prevented by anti-miR-124 transfection in mSOD1 MNs. After 2 days of differentiation, NSC-34 (wild type, WT) MNs and mSOD1 MNs were transfected with pre-miR-124 or anti-miR-124, respectively, for 12 h and maintained for additional 48 h. (**a**,**b**) Mitochondrial viability was measured by using MitoTracker™ Red staining. Protein levels of (**c**,**d**) mitofusin 2 (in green) and (**e**,**f**) DRP1 (in red) were evaluated by immunocytochemistry using specific antibodies. (**a**,**c**,**e**) Representative images are shown. Quantification of total fluorescence indicated in plots (**b**,**d**) and (**f**) was done in Image J software and normalized to the total cell number. Results are mean ± SEM from at least 4 independent experiments. ** *p* < 0.01, * *p* < 0.05 vs. WT MNs; ^##^ *p* < 0.01, ^#^ *p* < 0.05 vs. mSOD1 MNs; one-way ANOVA followed by multiple comparisons Bonferroni post-hoc correction. Scale bar: 20 μm. Drp1, dynamin related protein 1; MNs, motor neurons; mSOD1, MNs overexpressing G93A mutation in superoxide dismutase 1; WT, wild type.

Mitochondrial dysfunction in MNs is an event well described in several ALS in vitro models, including in our mSOD1 NSC-34 MN model [26]. As indicated in Figure 4a,b, both WT pre-miR-124 MNs and mSOD1 MNs showed decreased mitochondrial viability (*p* < 0.01 vs. untreated WT MNs). However, anti-miR-124 treatment in mSOD1 cells completely restored mitochondria vitality (*p* < 0.01 vs. mSOD1 MNs), reinforcing the importance of sustaining miR-124 expression levels within defined limits. Mitochondria vitality loss may derive from the decreased fusion and increased fission (Figure 4c–f; at least *p* < 0.05 vs. untreated WT MNs) in miR-124-enriched MNs, which is known to compromise mitochondria motility [39]. Such fragmentation of the mitochondrial network was previously reported in mSOD1 mice [40]. Intriguingly, the reestablishment of miR-124 expression levels led to the recovery of fusion–fission dynamics (at least *p* < 0.05 vs. mSOD1 MNs), therefore sustaining cellular quality control and cell bioenergetics efficiency [41,42].

Overall, these data indicate that the overexpression of miR-124 in WT MNs leads to MN dysfunctional mechanisms, as those found in mSOD1 MNs, and point to miR-124 deregulation as having a key role in disease-related MN degeneration in ALS, consequently supporting its potential as a biomarker. Reestablishment of functional MNs through the normalization of miR-124 levels validates such strategy in ALS therapeutics.

### 2.4. Microglia Polarization into a Pro-Inflammatory Phenotype by the Secretome from miR-124-Enriched mSOD1 MNs Is Prevented if the Mutated Cells Are Treated with Anti-miR-124

Deregulated intercommunication between neuronal and glial cells extensively contributes to ALS inflammatory milieu. Our previous studies showed that mSOD1 MN-derived exosomes enriched in miR-124 triggered microglia activation [25], and that the secretome from cortical ALS astrocytes caused microglia pathogenicity by paracrine signaling [43]. These findings emphasize the role of exosomes, as well as the secretome, in the homeostatic neuron–glia imbalance in ALS. Based on such data, we next assessed if the secretome from mSOD1 MNs treated with anti-miR-124 was able to prevent microglia phenotypic polarization induced by the pathological secretome. For that, microglia were isolated from the SC of 8-day-old WT mice and incubated for 4 h with the secretome from WT MNs, as well as from mSOD1 MNs, either untreated or treated with anti-miR-124. Incubation of microglia with WT MN secretome was intended to reproduce the normal steady state environment, and the timepoint of 4 h incubation was selected considering our prior data [25].

**Figure 5:**
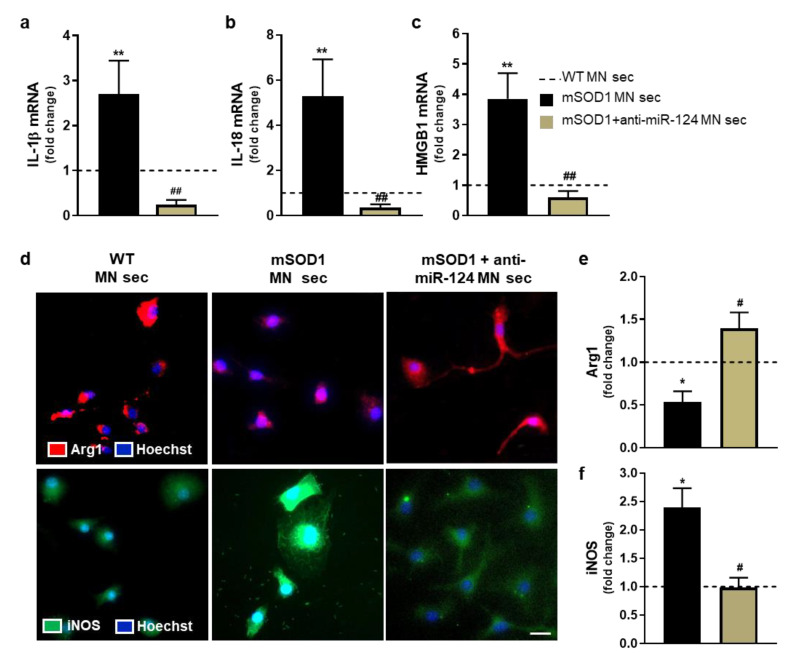
Secretome from mSOD1 MNs enriched in miR-124 activates the polarization of microglia from the SC of the WT mouse into a specific pro-inflammatory phenotype, while that from anti-miR-124 treated mSOD1 MNs sustain the homeostatic microglia subtype. Two days in vitro primary SC microglia were incubated with secretomes from untreated and anti-miR-124-treated mSOD1 MNs for 4 h. mRNA levels of (**a**) IL-1β, (**b**) IL-18, and (**c**) HMGB1 were evaluated by reverse transcriptase quantitative real-time PCR (RT-qPCR). β-actin was used as the reference gene. (**e**,**f**) iNOS and Arg 1 protein levels (in green and red, respectively) were evaluated by immunocytochemistry using specific antibodies and (**d**) representative images are shown. (**e**,**f**) Quantification of total fluorescence used Image J software, which was normalized to the total number of cells. Results are mean ± SEM from at least 3 independent experiments and are expressed as fold change vs. WT microglia incubated for 4 h with the WT MN secretome (dashed line). ** *p* < 0.01 and * *p* < 0.05 vs. cells incubated with the WT MN secretome; ^##^ *p* < 0.01 and ^#^ *p* < 0.05 vs. cells treated with mSOD1 MN secretome; one-way ANOVA followed by multiple comparisons Bonferroni post-hoc correction. Scale bar: 20 μm. SC, spinal cord; Arg1, arginase 1; HMGB1, high mobility group box protein 1; IL-1β, interleukin-1 beta; IL-18, interleukin 18; iNOS, inducible nitric oxide synthase; MN, motor neuron; mSOD1, G93A mutation in superoxide dismutase 1; sec, secretome; WT, wild type.

To be sure that the secretome from untreated and anti-miR-124-treated mSOD1 MN does not affect microglia viability, we assessed cell death by flow cytometry. As shown in Figure S2, no differences were observed for microglia incubated with the diverse MN-derived secretomes. In the next studies, the secretome from WT MNs was used as a control. We observed the overexpression of specific inflammatory-associated genes in microglia treated with mSOD1 MN secretome, such as interleukin (IL)-1β, IL-18, and high mobility group box protein 1 (HMGB1) (Figure 5a–c, at least *p* < 0.05 vs. WT MNs), but the polarized phenotype did not include alterations in tumor necrosis factor (TNF)-α or IL-10 mRNA expression (data not shown). However, expression of arginase 1 (Arg1) was reduced and that of inducible nitric oxide synthase (iNOS) elevated as quantified by immunostaining (Figure 5e,f, *p* < 0.05 vs. WT MNs). These results attest microglia polarization into a characteristic disease-associated subtype (ALS-DAM) due to mSOD1 MN paracrine signaling pathogenicity. When microglia were treated with the secretome from anti-miR-124 transfected mSOD1 MNs, such ALS-DAM phenotype was totally prevented (Figure 5a–f, at least *p* < 0.05 vs. mSOD1 MNs). This highlights that the regulation of miR-124 expression toward normal values may be a good strategy to return the activated microglia into the homeostatic state in the ALS disease condition. The increased number of Arg1-positive cells by the secretome from anti-miR-124 mSOD1 MNs may even dampen the anti-inflammatory characteristics of the MN-adjacent microglial cell. Overall, these data indicate that targeting miR-124 elevation in mutated MNs may be a promising strategy to avoid microglia polarization into the ALS-DAM phenotype and consequently, an effective approach to recover their neuroprotective phenotype.

### 2.5. Pathogenicity of Spinal Organotypic Cultures from Early Symptomatic mSOD1 Mice Is Minimized upon Addition of Secretome from Anti-miR-124-Treated mSOD1 MNs

To further investigate the homeostatic imbalance in the SC of mSOD1 mice and the potential neuroregeneration benefits by the secretome from mSOD1 MNs treated with anti-miR-124, we next explored reactive, immune, and neurodegenerative markers using 3D spinal organotypic cultures obtained at the early onset of the disease, i.e., from animals with 10–12-weeks-old [44,45]. We selected this ex vivo model since it preserves the basic structure and connective organization of a specific tissue or organ covering the gap between the in vitro and in vivo models [46], being accepted as a tool for translational biochemical research and pharmacology in ALS [47].

**Figure 6:**
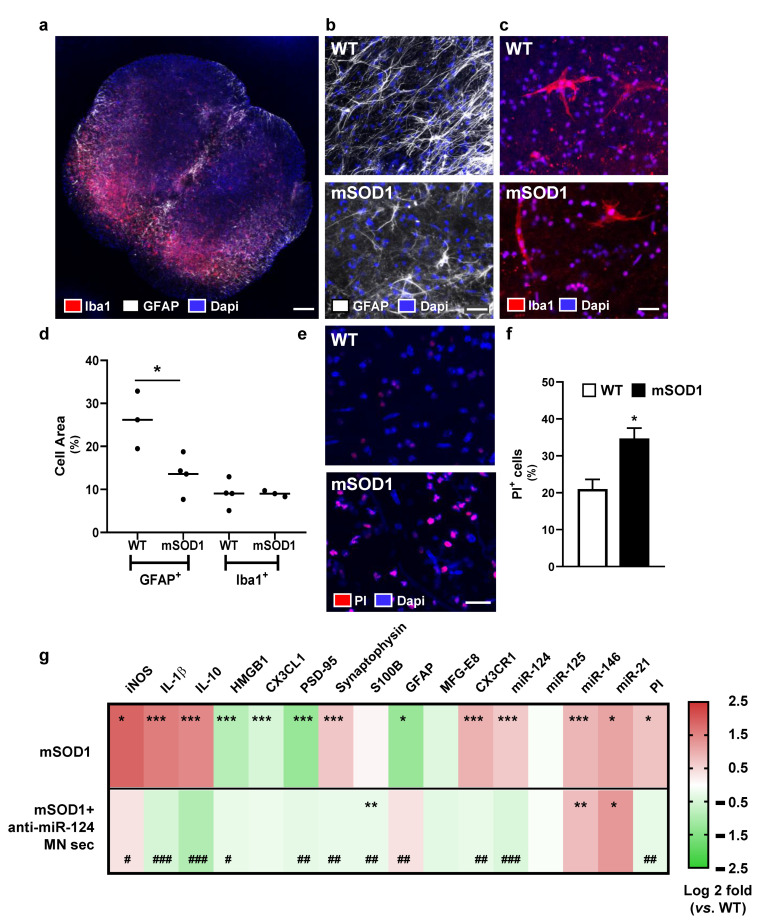
Spinal organotypic cultures from transgenic mSOD1 mice at early symptomatic stage show increased cell death, together with alterations in neuron–glia communication and dysregulation of inflammation-associated genes, which are suppressed by the secretome from anti-miR-124-treated mSOD1 MNs. Organotypic cultures were obtained from the spinal cord of 12-weeks old mice, either WT or TG. After 3 days in vitro, slices were incubated for additional 24 h with MN culture media. (**a**) Representative image of a transversal spinal cord slice stained for microglia and astrocytes. Nuclei are in blue (Dapi), microglia in red (Iba-1), and astrocytes in white (GFAP). Representative immunohistochemistry images for (**b**) GFAP (white, astrocytes) and (**c**) Iba-1 (red, microglia)-positive cells in WT and mSOD1 spinal cord organotypic cultures. (**d**) Percentage of area occupied by GFAP and Iba1 positive cells. (**e**) Representative images of ventral horn regions of WT and mSOD1 of SC organotypic slices stained for PI (red, dead cells) and co-stained for cell nuclei in blue (Dapi), followed by the (**f**) quantification of PI^+^ cells. Results are mean ± SEM from at least 3 independent experiments. * *p* < 0.05 vs. WT organotypic cultures using two-tailed unpaired Student’s *t*-test. (**g**) WT and mSOD1 organotypic cultures were incubated for 24 h with MN culture media or secretome from anti-miR-124 treated mSOD1 MNs (only in mSOD1 slices). mRNA expression of iNOS, IL-1β, IL-10, HMGB1, CX3CL1, PSD-95, synaptophysin, S100B, GFAP, MFG-E8, and CX3CR1, as well as of miR-124, miR-125b, miR-146a, and miR-21, was determined by reverse transcriptase quantitative real-time PCR (RT-qPCR), using β-actin as the reference gene (or SNORD110 for miRNAs). Heatmap shows gene expression fold change vs. WT slices incubated with MN culture media for 24 h. Results are mean ± SEM from at least 5 independent experiments. *** *p* < 0.001, ** *p* < 0.01, * *p* < 0.05 vs. WT slices, one-way ANOVA followed by multiple comparisons Bonferroni post-hoc correction. Scale bar: 200 μm (**a**) and 20 μm (**b**,**c**,**e**). CX3CL1, C-X3-C motif chemokine ligand 1; CX3CR1, C-X3-C motif chemokine receptor 1; GFAP, glial fibrillary acidic protein; HMGB1, high mobility group protein 1; IL-1β, interleukin-1 beta; IL-10, interleukin-10; iNOS, inducible nitric oxide synthase; MFG-E8, milk fat globule-EGF factor 8; MN, motor neuron; mSOD1, MNs overexpressing G93A mutation in superoxide dismutase 1; PI, propidium iodide; PSD-95, postsynaptic density protein 95 (Dlg4 gene); S100B, S100 calcium-binding protein B; sec, secretome; WT, wild type. (^##^ *p* < 0.01 and ^#^ *p* < 0.05, ^###^ *p* < 0.001).

SC from the lumbar region of mSOD1 and WT mice at 10–12-weeks-old was cut transversally with a thickness of 350 μm and maintained in culture during 3 DIV for trauma repairing process, followed by an additional period of 24 h with culture medium. To evaluate ALS pathology at this stage, we assessed astrocyte reactivity by evaluating the number of astrocytes staining for glial fibrillary acidic protein (GFAP) [48] and microglial activation by detecting the ionized calcium-binding adaptor molecule 1 (Iba1) marker [49]. A representative slice image from the WT SC organotypic culture is shown in Figure 6a, where GFAP and Iba-1 staining can be observed preferentially localized in the ventral region. Data obtained by immunohistochemistry at this timepoint (Figure 6b–d) indicate that GFAP^+^ and Iba-1^+^ cells occupy ~25% and 10%, respectively, of the area of the WT SC organotypic cultures. A decrease of approximately 10% in GFAP^+^ cells was observed in the mSOD1 cultures (*p* < 0.05 vs. WT mice), while no changes were noticed for microglia. Reduced expression of GFAP in the SC of mSOD1 mice was previously described in both pre- or symptomatic stages [3,50]. The number of propidium iodide (PI) positive cells, representing cell death [51], was found increased in the mSOD1 mouse cultures (Figure 6e,f; *p* < 0.05 vs. WT mice), most likely due to MN death, already recognized as an hallmark in the SC of mSOD1 mice at the symptomatic stage [3,45]. After identifying such neuropathological markers in the SC of mSOD1 mice at the early onset of the disease, we next evaluated if neuro-immune markers were exacerbated by the addition of the secretome from mSOD1 MNs, and whether they disappeared with that from anti-miR-124-treated mSOD1 MNs.

As indicated in Figure 6g and Table S4, we found a dysregulation of genes associated with neuro-immune imbalance in slices from mSOD1 mice, namely elevated levels of iNOS, IL-1β, and IL-10 (at least *p* < 0.05 vs. WT mice). Nevertheless, other genes associated with alarmin-associated paracrine signaling were unchanged (S100B) or even decreased (HMGB1), reinforcing the homeostatic imbalance and the decompensated neuro-immune inflammatory status. Compromised neuronal function may derive from the decreased chemokine (C-X3-C motif) ligand 1 (CX3CL1) levels and the concomitant elevation of its receptor CX3CR1 in microglia (*p* < 0.001 vs. WT mice), suggested to associate to neurodegeneration, or even to protection [52]. Increased levels of synaptophysin and decreased Dlg4, which encodes for PSD-95 (*p* < 0.001 vs. WT mice, for both), may be also due to compensatory mechanisms in deficient synaptic transmission. Downregulation of the GFAP gene in mSOD1 slices validated the immunohistochemistry data and the astrocyte aberrancies in this mouse model [4,53]. In addition, we observed only a slight decrease of milk fat globule-epidermal growth factor 8 (MFG-E8), which is associated to microglia phagocytosis and previously found reduced in this mice model at both pre- and symptomatic stages [3]. However, enhanced levels of inflamma-miRNAs were noticed, mainly of miR-124, miR-146a, and miR-21 (at least *p* < 0.05 vs. WT mice), once again attesting the ALS-associated immune-deregulation. All these spinal neuro-immune imbalance events culminated with an increased number of propidium iodide (PI) positive cells, indicating enhanced cell necrosis in our model.

Finally, we aimed to validate if the regularization of the miR-124 expression levels in the ALS MNs was translated into a secretome with neuroprotective and anti-inflammatory properties over the deregulated neuro-immune mediators that characterized the SC of the mSOD1 mice. We found that the increase of miR-146 and miR-21 was still sustained when compared to WT slices. However, the modulatory approach succeeded in regulating many of the transcripts (Figure 6g and Table S4). Indeed, the transcriptomic profile evidenced a decrease in the inflammatory-associated iNOS, IL-1β, and IL-10. Relatively to the neuronal synaptic function, PSD-95 and synaptophysin were restored to levels close to the WT organotypic cultures, as was the depressed GFAP. As expected, we observed a decrease of miR-124 levels by the secretome from mSOD1 MNs treated with anti-miR-124. Regularization of CX3CR1 was also obtained. The achieved profile evidence the presence of compensatory and adaptative mechanisms that may contribute to prevent the neuroinflammatory-associated disease progression in the SC of mSOD1 mice.

In sum, our results indicate that the downregulation of miR-124 in mSOD1 MNs toward physiological levels recovers neuronal vitality, prevents ALS-DAM polarization, and counteracts most of the abnormalities found in the SC organotypic cultures from mSOD1 animals at early disease onset. Data validate that the reduction of miR-124 levels in MNs, using direct cell targeting with anti-miR-124, or eventually anti-miR-124-based exosomes, in patients showing enriched miR-124 cargo in circulating exosomes, may result in a promising therapeutic tool to halt, or at least delay, ALS disease progression, mainly in cases of spinal origin. Finally, further studies are required to establish whether elevated levels of miR-124 in the circulation of ALS patients are associated to a poorer outcome, validating miR-124 as an important biomarker and therapeutic target in ALS pathology.

## 3. Discussion

We and other authors have found an elevation of miR-124 expression levels in ALS models and patients [3,21,22,23,24,25]. Our prior data revealed an increased expression of this miRNA in mSOD1 MN-like cells [25] and in the SC of symptomatic mSOD1 mice [3]. miR-124 is highly abundant in neurons [54,55] and its expression is fundamental for proper neuronal differentiation and function [14,15], as well as for homeostatic synaptic plasticity [34]. However, beneficial or harmful effects for miR-124 are differently addressed among studies. Its decrease was shown to attenuate NO production, while its upregulation was highlighted as increasing the risk of hypoxic injury and angiogenesis decrease, or even to be protective in SC injury [56,57]. In addition, miR-124 dysregulation was linked to the development of neurodegenerative disorders [31] and to several immune-system related pathways [58]. On the other hand, potential benefits of miR-124 upregulation were referred in Alzheimer’s disease through negative regulation of BACE1 expression [59]. Therefore, upregulation and downregulation of miR-124 may exert different properties and biological effects depending on the disease experimental models, the type of neurons and their regional identity. Given the presence of miR-124 elevated levels in ALS MNs, this study intended to explore whether such upregulation was associated to compensatory mechanisms or to pathogenicity.

We started by assessing if different expression levels of miR-124 regulated MN survival. For that, we used pre-miR-124 in WT MNs and anti-miR-124 in mSOD1 MNs, which originally have upregulated miR-124 levels [25]. The Authors showed that it was not only present in MNs, but also included in their secretome-derived exosomes, attesting its passive release into the extracellular vesicles [60]. Thus, attempts to modulate cellular miRNA expression levels are expected to be translated not only into autocrine, but also into paracrine signaling events to neighboring cells. In the case of miR-124, its increased levels were associated to MN proneness to early apoptosis and MN dysfunction in ALS, though it may also compromise neuro-immune homeostatic balance. Recently, upregulation of miR-124 in exosomes from spinal MNs and in CSF from ALS patients was proposed as a potential biomarker based on its correlation with disease severity [24].

In this study, we confirmed that both mSOD1 MNs and treatment with pre-miR-124 in WT MNs triggered a loss on cell viability, which was prevented when we used anti-miR-124 in mSOD1 MNs, while it also influenced its direct cargo in exosomes. We next observed that changes in the cellular content of miR-124 similarly caused alterations in other inflamma-miRNAs, including those in MN-derived secretome. Moreover, when increased, miR-124 led to decreased expression of miR-146a and miR-21 in both cells and their secretome. However, despite being elevated in cells overexpressing miR-124, decreased levels of miR-125b were observed in the secretome derived from mSOD1 MNs, independently of being modulated or not with anti-miR-124, indicating that the mutated cells actively retain or differently metabolize miR-125b. In fact, it was already demonstrated that fine regulation of this miRNA is essential for functional cell recovery and correct axonal regeneration after SC injury [61]. Accordingly, miR-125b preservation in dysfunctional mSOD1 MNs may constitute a self-repair strategy given its role in sustaining neuronal differentiation, neurite outgrowth, and synaptic function and structure [62]. The recovery of miR-146a levels in mSOD1 MNs and roughly in their derived secretome using anti-miR-124 approach may have beneficial relevance. Indeed, miR-146a is associated to anti-inflammatory processes and its reduced expression implicated in astrocyte aberrancies and neurotoxic events in the ALS mouse model [43]. Similarly, the downregulation of miR-21 in cells and their secretome whenever miR-124 was elevated, and its recovery when mSOD1 MNs were transfected with anti-miR-124, may have important immunomodulatory consequences in glial cells. Indeed, miR-21 has a crucial anti-inflammatory role in neuron–microglia communication, influencing microglial activation via exosomes and secretome [63,64]. Notably, miR-21 upregulation in neurons may contribute to axonal regeneration, as well [65].

In previous studies, miR-124, alone or associated with miR-9, was indicated as favoring dendritic branching [30,66]. In this study, enhanced primary length and reduced branching was observed when miR-124 was increased, and the opposite was obtained by its downregulation. In a recent paper, mutant SOD1 MNs resistant to disease development showed enhanced axonal outgrowth and dendrite branching [67], which may represent a compensatory mechanism in cytoskeletal regulation, though we only observed for the neurite length. Intriguingly, the intermediate filament vimentin that is associated to neurite outgrowth [20] and a target of miR-124 [20,38], showed to be decreased when miR-124 was upregulated, but normal with anti-miR-124 modulation. Other factors accounting for reduced branching by miR-124 upregulation may derive from miR-146a downregulation and its known influence on the cytoskeleton actin through ROCK1 [68]. Considering that axonal deterioration is one of the earliest events of MN degeneration [69] alterations in neuritic tree in mSOD1 MNs and by miR-124 upregulation may then precede neurodegeneration in ALS mice [67]. Reasons for miR-124 detrimental effects, in contrast with studies suggesting benefits, may reside on the functional differences of MNs relatively to neurons. When from spinal origin, as in this study, they were shown to respond to acetylcholine instead of glutamate, and to be involved in direct stimulation of muscles to contract [70]. In our case, gains by reducing miR-124 levels toward control ones should consider its role in MN regeneration [71] and in the regulation of spinal MN subtypes [72].

Trans-synaptic signaling unbalance dependent on miR-124 increased expression is reflected in the upregulation of the pre-synaptic marker synaptophysin and downregulation of the post-synaptic PSD-95, either in the mutated cells or in the pre-miR-124-treated WT MNs. Defects in pre-synaptic or post-synaptic terminals have been considered hallmarks in ALS pathology [6], with a reduction in the number of postsynaptic densities in ALS patients [73]. Data validate that miR-124 should be maintained in restricted expression levels to sustain homeostatic synaptic plasticity [34]. Pathological axonal transport is also commonly observed in different MN diseases and considered to be an early event of MN demise in ALS [74,75]. We observed that MNs with upregulated levels of miR-124 show a decreased anterograde transport and an increased retrograde transport, based on kinesin downregulation and dynein upregulation, respectively, which were also previously observed in transgenic ALS mice [76,77]. Since anti-miR-124 led to the restoration of normal ones in mSOD1 MNs, we may hypothesize that the inhibition of anterograde transport in mSOD1 MNs may be a consequence of miR-124 upregulation. Tightly associated with the axonal transport impairment is the mitochondrial dysfunction, again a hallmark in ALS pathology [78,79,80], that was similarly observed in these same cells before [4,26]. Here, mitochondrial dysfunction in mSOD1 MNs was associated with miR-124 upregulation. This is not without precedent, as previous studies reported that miR-124 regulates mitochondrial activity and motility in primary MNs [20]. Taken together, miR-124 and mitochondrial function thus seem to be associated. Decreased levels of mitofusin 2 and elevation of the fission protein dynamin-related protein 1 (DRP1) by upregulated miR-124 suggests a direct influence on the mitochondrial dynamic deregulation, considering that it was prevented by anti-miR-124 treatment. In fact, a similar profile of mitochondrial deregulation was described in the SC of mSOD1 mice at the onset of the disease [40]. Moreover, it was recently demonstrated that the peptide inhibitor P110, known to block the association of DRP1/mitochondrial fission 1 protein (Fis1) with mitochondria, was able to restore mitochondrial dynamics, as well as to enhance motor activity and life span in the mSOD1 mouse model [81]. Such features reinforce the therapeutic potential of blocking mitochondrial fission and the resultant fragmentation by delivering anti-miR-124. This strategy was shown to promote mitophagy and spinal neuroprotection in the ischemia–reperfusion injury condition [82].

We have demonstrated that miR-124-enriched exosomes from mSOD1 MNs induced N9-microglia overactivation, suggesting that the transference of this miRNA may have important detrimental paracrine signaling effects to the neighboring cells [25]. Release of miR-124 from neurons seems to also be associated with high density lipoproteins [83], accounting for an important signaling communication between these two cell types. Considering that communication mediated by soluble factors has been implicated in ALS pathological dissemination [84], the next step was to explore whether the regulation of miR-124 toward normal values in mSOD1 MNs contributed to paracrine signaling improvement and inhibition of microglia activated phenotypes via contact-independent mechanisms [85]. We tested the neuronal-mediated secretome influence on spinal microglia obtained from mice pups (8 days-old), an aspect never tested before in this context.

Our data support that the mSOD1 MN secretome determines a shift from steady-state microglia immunophenotype into a pro-inflammatory subtype as manifested by the increased expression of IL-1β, IL-18, and HMGB1 genes, together with enhanced iNOS and decreased Arg1, a profile earlier identified by us in N9-microglia overexpressing mSOD1 [86]. Data also agree with Gugliandolo et al. [87] relatively to inflammasome priming associated to IL-1β and IL-18 activation in a SOD1 mouse model. Concerning the increased gene expression of HMGB1, it has been coupled to stress-induced inflammasome activation in microglia [88]. Such microglia polarization was prevented when we achieved lower levels of miR-124 in the secretome after mSOD1 MN treatment with anti-miR-124, which then sustained the microglia surveillant homeostatic phenotype and reinforced the relevance of keeping controlled levels of miR-124 in mSOD1 MNs.

To further explore the benefits of the MN anti-miR-124 modulatory strategy, we tested its efficacy in counteracting the neuro-immune imbalance that characterize the SC of mSOD1 mice at early symptomatic stage, by using organotypic cultures. This 3D model preserves tissue architecture and functional cell crosstalk as in vivo [46]. To note, however, that it was a challenge to deal with the low yield of the collected specimens and their preservation-associated difficulties linked to an increased proneness of the samples to neurodegeneration when obtained from adult animals, and not from 7-day-old mice [89].

The considerable amount of necrotic cell death (~20%) found in WT SC organotypic cultures may then relate to the age of the animals and mechanical trauma caused by the tissue chopper cutting. Levels of cell necrosis and impairment of synaptic markers were found enhanced in mSOD1 SC organotypic cultures. The increase of synaptophysin along with the decrease of PSD95 suggest MN loss, as we observed with the mSDO1 MN-cell line. Low levels of CX3CL1 in mSOD1 slices also suggest neurodegeneration aggravated by neuron–glia communication failure [90]. Decreased levels of GFAP mRNA may be attributed to astrocyte aberrant phenotype in mSOD1 organotypic cultures, as observed in the SC of mSOD1 mice at disease onset [3]. Similarly, heterogeneous pro-/anti-inflammatory associated genes and miRNAs (i.e., elevated iNOS, IL-1β, IL-10, miR-124, miR-146, and miR-21), together with cell increased proliferation and upregulated synaptophysin, TREM2, and CX3CR1 in the SC organotypic cultures from mSOD1, additionally validate the existence of a deregulated inflammatory status [3,9].

The strategy of suppressing elevated miR-124 levels in the secretome, by treating mSOD1 MNs overexpressing miR-124 with its inhibitor, mostly counteracted the pathological phenotype and the disturbed homeostatic imbalance of the SC organotypic cultures from the transgenic mice upon secretome addition. We highlight the decreased PI-associated cell demise, the regularization of inflammatory associated markers and of GFAP expression, as well as the recovery of synaptic balance and the downregulation of miR-124 as improvements when miR-124 expression is downregulated. To also emphasize that miR-124 targeting did not influence the levels of miR-21 and miR-146a that were maintained upregulated, constituting a drive force to positively control neuro-immune status and cell survival, based on their anti-inflammatory effects [91].

Taken as a whole, our results support miR-124 upregulation in MNs and in their secretome as being involved in neurodegeneration and deregulated cell-to-cell communication, thus contributing to ALS pathogenicity. Furthermore, the study supports potential anti-miR-124 intervention in patient subpopulations with elevated circulating levels, its value as a biomarker for diagnosis and patient stratification, while also provides new perspectives for target-driven therapeutic strategies, preferentially at ALS early stages.

## 4. Materials and Methods

### 4.1. Culture and Transfection of NSC-34 MN-Like Cell Line

Mice NSC-34 MN cell line stably transfected with human superoxide dismutase 1 (hSOD1), either wild-type (WT) or carrying a glycine to alanine point mutation at residue 93 (G93A), here designated as mSOD1, was used as usual in our lab [26].

Briefly, cells were plated in poly-d-lysin 12-well tissue culture plates (1.3 × 10^4^ cells/cm^2^) and grown in proliferation media [DMEM high glucose w/o pyruvate, 10% fetal bovine serum (FBS, Life Technologies, MA, USA), 1% penicillin/streptomycin (Pen/Strep, Sigma-Aldrich, St. Louis, MO, USA), and 0.1% geneticin sulfate 418 (G418, Merck) for cell selection]. After 48 h, differentiation was induced by changing medium to DMEM-F12 (Life Technologies, Waltham, MA, USA) plus 1% FBS, 1% nonessential amino acids (NEAA, Merck, Darmstadt, Germany), 1% Pen/Strep, and 0.1% G418. After 1 day in vitro (1 DIV), mSOD1 MNs were transfected with 15 nM of Anti-miR^TM^ 124 Inhibitor (#AM10691, Ambion^®^), while WT MNs were transfected with 15 nM of Pre-miR^TM^ 124 Precursor (#PM10691, Ambion^®^, Thermo Fisher Scientific). The mature miR-124 sequence was 5′-UAAGGCACGCGGUGAAUGCC-3′ (has-miR-124-3p). Then, the oligonucleotides were mixed with X-tremeGENE™ HP DNA Transfection Reagent (Sigma-Aldrich) in a proportion 2:1 and diluted in Opti-MEM™. Cell transfection occurred during 12 h. Fresh differentiation medium was added and the cells were incubated for additional 48 h. Cells were collected after 4 DIV, a timepoint selected based on our prior studies in mSOD1 MNs showing SOD1 accumulation, mitochondrial dysfunction, and miR-124 elevated levels in mSOD1 MNs [25,26]. Cells were collected for immunostaining or RNA isolation. Collection of non-transfected WT/mSOD1 and anti-miR-124 mSOD1 MN secretomes was performed for miRNA evaluation or incubation in the subsequent experimental models, as described below. The non-transfected WT and mSOD1 MNs, incubated with Optimem, were used as controls. In parallel, WT and mSOD1 MNs were treated with X-tremeGENE™ HP DNA Transfection Reagent diluted in Opti-MEM™ (mock controls). To ensure that the results obtained by cell transfection were due to miR-124 specificity, we also transfected WT cells with 15 nM of Pre-miR Negative Control and mSOD1 ones with 15 nM Anti-miR Negative Control (Ambion^®^). We observed no differences between non-treated, mock controls, and the respective Pre-miR Negative Control, as well as the Anti-miR Negative Control, validating the miR-124 specificity (Figure S1).

### 4.2. Mice Models

Transgenic B6SJL-TgN (SOD1-G93A)1Gur/J males (Jackson Laboratory, no. 002726) overexpressing the hSOD1G93A (mSOD1) [44] and B6SJLF1/J non-transgenic wild type (WT) females were purchased from The Jackson Laboratory (Bar Harbor, ME, USA). The maintenance and handling took place at the Instituto de Medicina Molecular João Lobo Antunes animal house facilities from Faculty of Medicine, University of Lisbon, Portugal, where the colony was established. Mice were maintained on a background B6SJL by breeding SOD1G93A (mSOD1) transgenic males with non-transgenic females. Transgenic SOD1-G93A mice were compared to aged-matched WT mice. All animals were maintained on 12 h light/12 h dark cycle and received food and water ad libitum. Average number of animals per cage was 4 to 5. The present study was performed in accordance with the European Community guidelines (Directives 86/609/EU and 2010/63/EU, Recommendation 2007/526/CE, European Convention for the Protection of Vertebrate Animals used for Experimental or Other Scientific Purposes ETS 123/Appendix A) and Portuguese Laws on Animal Care (Decreto-Lei 129/92, Portaria 1005/92, Portaria 466/95, Decreto-Lei 197/96, Portaria 1131/97). All the protocols used in this study were approved by the Portuguese National Authority (General Direction of Veterinary) and the Ethics Committee of the IMM. According with the 3R’s principle, every effort was made to minimize the number of animals used and their suffering.

#### 4.2.1. Primary Spinal Microglia Cultures and Treatments

Mixed glial cultures were prepared from the SC of 7-day-old WT and SOD1G93A mice. Our procedure was based in the method described by Saura et al. [92] to isolate microglia from rat cerebral cortices with minor adaptions for mice [27]. Cells were plated on uncoated tissue culture plates and maintained in culture medium [DMEM-Ham’s F-12 medium supplemented with 2 mM L-glutamine (Merck), 1 mM sodium pyruvate (Merck), 1% NEAA, 10% FBS, and 1% of Antibiotic-Antimycotic solution (Sigma-Aldrich)] at 37 °C, 5% CO_2_. Medium was changed every 7 days. After 21 DIV, microglia were isolated by mild trypsinization to detach the upper layer of astrocytes. The remained attached spinal microglia were maintained in culture for 2 DIV. Secretomes from WT MNs, mSOD1 MNs, and anti-miR-124-treated mSOD1 MNs were incubated with microglia for 4 h. Cells incubated with WT MN were used as controls. Cells were collected for immunostaining or RNA isolation, as described in the next sections.

#### 4.2.2. Spinal Cord Organotypic Cultures and Treatments

SC organotypic cultures from the mSOD1 mice were used to explore ALS homeostatic imbalance and cell pathological features relatively to matched controls. These cultures preserve the basic structure and connective organization of a specific tissue or organ of interest [46], allowing interactions and paracrine signaling between neurons and glial cells [93]. WT and mSOD1 early symptomatic animals (~10–12 weeks old mice) were used in our experiments. Animals were anesthetized with inhalant isoflurane prior to sacrifice. The protocol was based on the one established for the SC of mice pups [89] with minor adaptations. Dissected lumbar portions of the SC were embedded in ice-cold HBSS medium (with 6 mg/ml glucose and 5% Pen/Strep) to simulate in vivo pH and osmotic balance and maintain the tissue consistency. Transversal SC slices of 350 µm were obtained by using a McIlwain Tissue Chopper^®^ (Gomshall, Surrey, UK). A set of 4 slices were placed in the upper face of the insert membrane (BD Falcon, NJ, USA) and cultured on the surface of 0.4 µm pores inserts membrane. The inserts were then placed in 6-well plates with culture medium (Neurobasal medium plus 2% B27, 1.33 % glucose, 1.5% Pen/Strep, and 1% L-glutamine) during 3 DIV for trauma cut recovery. Fresh culture medium was added each 24 h and SC organotypic cultures were maintained at 37 °C in a humidified atmosphere of 5% CO_2_ in the air-liquid interface. At 3 DIV, WT and mSOD1 SC organotypic cultures were incubated with MN secretome, as in Section 4.2.1, for 24 h. Organotypic cultures from WT mice exposed to MN culture media were used as controls. After this time, slices were collected for assessment of cell necrosis, immunostaining, or RNA isolation, as described in the next sections. No change in color and transparency was detected during the culture under the light microscope.

### 4.3. RT-qPCR

Total RNA from NSC-34 and spinal microglia cells were isolated using TripleXtractor Reagent (GRiSP, Porto, Portugal) [43]. Concerning the NSC-34 derived secretome, total RNA (including miRNAs) was extracted using miRNeasy Serum/Plasma Advanced kit (QIAGEN, Valencia, CA, USA) according to manufacturer’s instructions. For SC organotypic slices, we used a Pellet Mixer (VWR Life Science, EUA) for sample homogenization and the Trizol^®^ Reagent (Life Technologies) for RNA isolation [3]. Then, RNA was quantified using NanoDrop ND100 Spectrophotometer (NanoDrop Technologies, Wilmington, USA). For miRNA expression, cDNA conversion for miRNA was performed with the miRCURY LNA^TM^ RT Kit (QIAGEN), using 5 ng of total RNA for cells and 10 ng for secretome. Then, the PowerUp™ SYBR™ Green Master Mix (Applied Biosystems, Life Technologies) was used in combination with pre-designed primers listed in Table S1 to perform the RT quantitative PCR (RT-qPCR). The reaction conditions consisted of polymerase activation/denaturation and well-factor determination at 95 °C for 10 min, followed by 50 amplification cycles at 95 °C for 10 s and 60 °C for 1 min (ramp-rate of 1.6°/s). A melting curve analysis was done after the amplification to verify specificity. SNORD110 and RNU1A1 were used as endogenous controls.

For gene expression, Xpert cDNA Synthesis Mastermix kit (GRiSP) was used to convert RNA. Later, RT-qPCR was performed by using Xpert Fast SYBR Mastermix BLUE kit (GRiSP). The primer sequences used are listed in Table S2. Each amplification product was obtained under the follow optimized conditions: 50 °C for 2 min, 95 °C for 2 min, followed by 45 amplification cycles at 95 °C for 5 s and 62 °C for 30 s. A melting curve analysis was done after the amplification to verify the specificity. β-actin was used as the endogenous control to normalize each gene expression level.

Relative mRNA/miRNA concentrations are represented as fold change and determined by the 2-ΔΔCT method [3]. Quantification of target miRNAs was made in comparison to the geometric average of the two reference controls. In addition, we used the synthetic RNA template spike-in (UniSp6) as a positive control to ensure the quality of the reaction and subsequent evaluations. All samples were measured in duplicate. cDNA synthesis was performed in a thermocycler (Biometra^®^, Göttingen, Germany) and the RT-qPCR was accomplished on QuantStudio 7 Flex Real-Time PCR System (Applied Biosystems).

### 4.4. Cell Death by Nexin Assay

We analyzed cell viability as described [4]. Briefly, cells were trypsinized and added to those detached into the extracellular media. A centrifugation was performed, and the pellet then resuspended in 1% bovine serum albumin (BSA) in PBS. The Nexin Reagent^®^ (Millipore) was added to the samples according to manufacturer’s instructions and analyzed on Guava easyCyte 5HT Base System Flow Cytometer (Guava Nexin^®^ Software module, Millipore). Phycoerythrin-conjugated Annexin V (Annexin V-PE) and 7-amino-actinomycin D (7-AAD) were used to determine viable (Annexin V-PE and 7-AAD negative), early apoptotic (Annexin V-PE positive and 7-AAD negative), and late apoptotic/necrotic (Annexin V-PE and 7-AAD positive) cells.

### 4.5. Immunocytochemistry

We evaluated the neurite extension and the ramification number using anti-βIII tubulin, the levels of mitochondrial fission with Drp1 and fusion by mitofusin 2 in NSC-34 MNs, together with pro-/anti-inflammatory-associated markers, such as iNOS and Arg1 in spinal microglia.

NSC-34 MNs and spinal microglia plated on coverslips were fixed with 4 % (*w*/*v*) of paraformaldehyde (PFA) and the immunostaining performed as published for MNs and microglia, respectively [4,94]. Cells were incubated with 0.2% Triton X-100 in PBS for cell permeabilization during 20 min, 3% BSA in PBS for blocking during 30 min, and primary antibodies (listed in Table S3) overnight at 4 °C. Then, incubation with species-specific fluorescent secondary antibodies, referred in Table S3, occurred for 2 h at room temperature. Cell nuclei were stained with Hoechst 33258 dye and coverslips were mounted onto uncoated slides with PBS-glycerol (1:1). Fluorescence was visualized in an AxioCam HR camera adapted to an AxioScope A1^®^ microscope and Zen 2012 (blue edition, Zeiss) software. Merge images of UV and fluorescence from at least 6 random fields were acquired with 40· magnification per condition. Neurite extension and number were assessed by immunofluorescence detection of the cytoskeletal protein βIII-tubulin, according with the immunocytochemistry assay described in Section 4.5. Using the ImageJ plugin NeuronJ, the primary branches, emanating directly from the soma, as well as the secondary/tertiary ones sorting primary/secondary neurites were manually traced [95,96]. Total fluorescence intensity of Drp1, Mitofusin 2, iNOS, and Arg1 was quantified by ImageJ software and normalized to the total cell nuclei number.

### 4.6. Immunohistochemistry

We evaluated microglial and astrocytic content through the quantification of the expression of cell specific markers, namely Iba1 for microglia and GFAP for astrocytes. Slices were washed with PBS and immediately fixed with 4% PFA for 1 h at room temperature for the immunostaining procedure. Slices were washed one more time with PBS and removed from the insert into glass slides, followed by blocking for 3 h at room temperature using 2% heat-inactivated horse serum, 10% FBS, 1% BSA, 0.25% Triton X-100, and 1 nM HEPES. Slices were subsequently incubated with primary antibodies (indicated in Table S3) at 4 °C for 48 h. Then, slices were washed 3 times for 20 min with 0.01% Triton X-100 in PBS solution and incubated with secondary antibodies (indicated in Table S3) for 24 h at 4 °C. Slices were washed in the same conditions and cell nuclei stained with DAPI (1:1000 in PBS) for 5 min. Slices were additionally washed 3 times with PBS. Lastly, slices were mounted with one drop of Fluoromount-G and one coverslip was applied for confocal microscopy. Images of at least 5 ventral horn fields per condition (at least 3 different experiments) were obtained with 40· magnification. Fluorescence images were acquired in a Leica DMi8-CS inverted microscope with Leica LAS X software and the different z-stacks were merged and analyzed with Fiji software.

### 4.7. Mitochondrial Viability

NSC-34-MN cells were incubated for 30 min at 37 °C with 500 nM of MitoTracker^TM^ Red CMXRos solution (Invitrogen, Oregon, EUA) to stain viable mitochondria, and then fixed with PFA at 4% [26]. Cell nuclei were stained with Hoechst 33258 dye. Images were acquired as above for immunocytochemistry. Total fluorescence intensity of Mitotracker Red was quantified by ImageJ software and normalized to the total number of cell nuclei.

### 4.8. Cell Death Determination by Propidium Iodide Uptake

Necrotic cell-like death was determined by monitoring the cellular uptake of the fluorescent dye PI. After incubation with NSC-34-MN secretome, unpermeabilized slices were incubated with the PI solution for 15 min at 37 °C in a humidified atmosphere of 5% CO_2_ and in absence of light. We used a stock PI solution (0.5 mg/mL) diluted 1:20 in the culture medium. Slices were then washed with PBS and fixed with PFA 4% for 1 h at room temperature and protected from light. Then, they were stained with DAPI dye (Merck) and mounted with Fluoromount-G (Southern Biotech, Birmingham, AL). Fluorescence images were acquired by using the Leica DMi8-CS inverted microscope with Leica LAS X software, with 40· magnification. The different z-stacks were merged and analyzed in Fiji software. Images of at least 5 ventral horn fields per condition were obtained. A threshold of mean fluorescence ≥ of 100 pixels was established for PI-positive cells. Moreover, particles between 3 and 50 μm^2^ size co-stained with blue (DAPI) and red (PI) were considered dead cells.

### 4.9. Statistical Analysis

Statistical analysis was performed using GraphPad Prism 8.0.1 (GraphPad Software, San Diego, CA, USA). Results of at least three different experiments are presented as mean values ± SEM. Results of transfected NSC-34-MN cells and their derived secretome are expressed relatively to non-transfected WT MNs. Data from spinal microglia incubated with the secretome from non-transfected or transfected mSOD1 MNs were compared with microglia incubated with the WT MN secretome. Data from mSOD1 organotypic slices incubated with the non-transfected or transfected mSOD1 MN secretome was compared with WT slices exposed to MN culture media. One-way ANOVA followed by multiple comparisons Bonferroni post-hoc correction was applied. Values of *p* < 0.05 were considered statistically significant.

## 5. Conclusions

This study presents evidence that miR-124 levels above the normal ones in nSOD1 MNs are implicated in cell pathological alterations and in dysregulated signaling events that lead to microglia activation and neuro-immune impaired interactions. At the MNs, upregulated miR-124 was implicated in cell demise, synaptic signaling dysregulation, morphological changes in cell arborization, axonal transport deficiencies, and mitochondrial dysfunction, concomitant with miR-125b, miR-146a, and miR-21 signature changes in cells and in their secretome. The predominant origin of miR-124 in neurons and its enrichment in exosomes concur for its applicability as a promising and disease-stage biomarker in circulating fluids, such as the CSF and the blood.

The targeting of miR-124 toward normal levels is also a therapeutic promise in ALS if we take into consideration that such strategy increases MN rate survival, vitality, and function, and that neuroprotection extends to synapse, mitochondria, and axonal transport dynamics. Additionally, to emphasize that once regulated in ALS MNs, miR-124 also accounts to a MN-derived secretome exerting protection to the cell environment. This is the case of the spinal microglia in ALS showing heterogeneous and complexed polarized phenotypes that switches to a steady state phenotype by the influence of such controlled MN-derived secretome. By regulating neuroinflammation, the regularization of miR-124 expression levels in ALS MNs and subsequent paracrine signaling may halt, or at least delay, disease progression. In a more complex context, as that of organotypic cultures of the SC of mSOD1 mice at early after disease onset, secretome from anti-miR-124-treated MNs by reversing most of the pathological events associated to immune dysregulation shows promise as a viable strategy to reduce inflammatory response and pathogenicity in ALS disease. In this case, exosomes as tools for anti-miR-124 delivery may emerge in ALS regenerative medicine, using new administration routes as the retro-orbital vein or intranasal delivery, mainly in spinal disease patients with enriched circulating levels of miR-124.

## Supplementary Materials

The following are available online at https://www.mdpi.com/article/10.3390/ijms22116128/s1.

## References

[1] Khalid S.I., Ampie L., Kelly R., Ladha S.S., Dardis C. (2017). Immune Modulation in the Treatment of Amyotrophic Lateral Sclerosis: A Review of Clinical Trials. Front. Neurol..

[2] Brites D., Vaz A.R. (2014). Microglia centered pathogenesis in ALS: Insights in cell interconnectivity. Front. Cell Neurosci..

[3] Cunha C., Santos C., Gomes C., Fernandes A., Correia A.M., Sebastião A.M., Vaz A.R., Brites D. (2018). Downregulated Glia Interplay and Increased miRNA-155 as Promising Markers to Track ALS at an Early Stage. Mol. Neurobiol..

[4] Gomes C., Cunha C., Nascimento F., Ribeiro J.A., Vaz A.R., Brites D. (2019). Cortical Neurotoxic Astrocytes with Early ALS Pathology and miR-146a Deficit Replicate Gliosis Markers of Symptomatic SOD1G93A Mouse Model. Mol. Neurobiol..

[5] Alrafiah A.R. (2018). From Mouse Models to Human Disease: An Approach for Amyotrophic Lateral Sclerosis. In Vivo.

[6] Ferraiuolo L., Kirby J., Grierson A.J., Sendtner M., Shaw P.J. (2011). Molecular pathways of motor neuron injury in amyotrophic lateral sclerosis. Nat. Rev. Neurol..

[7] Bento-Abreu A., Van Damme P., Van Den Bosch L., Robberecht W. (2010). The neurobiology of amyotrophic lateral sclerosis. Eur. J. Neurosci..

[8] Van Damme P., Robberecht W., Van Den Bosch L. (2017). Modelling amyotrophic lateral sclerosis: Progress and possibilities. Dis. Model Mech..

[9] Brites D. (2020). Regulatory function of microRNAs in microglia. Glia.

[10] Butovsky O., Jedrychowski M.P., Cialic R., Krasemann S., Murugaiyan G., Fanek Z., Greco D.J., Wu P.M., Doykan C.E., Kiner O. (2015). Targeting miR-155 restores abnormal microglia and attenuates disease in SOD1 mice. Ann. Neurol..

[11] Campos-Melo D., Droppelmann C.A., He Z., Volkening K., Strong M.J. (2013). Altered microRNA expression profile in Amyotrophic Lateral Sclerosis: A role in the regulation of NFL mRNA levels. Mol. Brain.

[12] Foggin S., Mesquita-Ribeiro R., Dajas-Bailador F., Layfield R. (2019). Biological Significance of microRNA Biomarkers in ALS-Innocent Bystanders or Disease Culprits?. Front. Neurol..

[13] Figueroa-Romero C., Hur J., Lunn J.S., Paez-Colasante X., Bender D.E., Yung R., Sakowski S.A., Feldman E.L. (2016). Expression of microRNAs in human post-mortem amyotrophic lateral sclerosis spinal cords provides insight into disease mechanisms. Mol. Cell Neurosci..

[14] Sun Y., Luo Z.M., Guo X.M., Su D.F., Liu X. (2015). An updated role of microRNA-124 in central nervous system disorders: A review. Front. Cell Neurosci..

[15] Neo W.H., Yap K., Lee S.H., Looi L.S., Khandelia P., Neo S.X., Makeyev E.V., Su I.H. (2014). MicroRNA miR-124 controls the choice between neuronal and astrocyte differentiation by fine-tuning Ezh2 expression. J. Biol. Chem..

[16] Ponomarev E.D., Veremeyko T., Barteneva N., Krichevsky A.M., Weiner H.L. (2011). MicroRNA-124 promotes microglia quiescence and suppresses EAE by deactivating macrophages via the C/EBP-alpha-PU.1 pathway. Nat. Med..

[17] Ponomarev E.D., Veremeyko T., Weiner H.L. (2013). MicroRNAs are universal regulators of differentiation, activation, and polarization of microglia and macrophages in normal and diseased CNS. Glia.

[18] Morel L., Regan M., Higashimori H., Ng S.K., Esau C., Vidensky S., Rothstein J., Yang Y. (2013). Neuronal exosomal miRNA-dependent translational regulation of astroglial glutamate transporter GLT1. J. Biol. Chem..

[19] Zhou F., Zhang C., Guan Y., Chen Y., Lu Q., Jie L., Gao H., Du H., Zhang H., Liu Y. (2018). Screening the expression characteristics of several miRNAs in G93A-SOD1 transgenic mouse: Altered expression of miRNA-124 is associated with astrocyte differentiation by targeting Sox2 and Sox9. J. Neurochem..

[20] Yardeni T., Fine R., Joshi Y., Gradus-Pery T., Kozer N., Reichenstein I., Yanowski E., Nevo S., Weiss-Tishler H., Eisenberg-Bord M. (2018). High content image analysis reveals function of miR-124 upstream of Vimentin in regulating motor neuron mitochondria. Sci. Rep..

[21] Marcuzzo S., Bonanno S., Kapetis D., Barzago C., Cavalcante P., D’Alessandro S., Mantegazza R., Bernasconi P. (2015). Up-regulation of neural and cell cycle-related microRNAs in brain of amyotrophic lateral sclerosis mice at late disease stage. Mol. Brain.

[22] Parisi C., Arisi I., D’Ambrosi N., Storti A.E., Brandi R., D’Onofrio M., Volonte C. (2013). Dysregulated microRNAs in amyotrophic lateral sclerosis microglia modulate genes linked to neuroinflammation. Cell Death Dis..

[23] Waller R., Wyles M., Heath P.R., Kazoka M., Wollff H., Shaw P.J., Kirby J. (2017). Small RNA Sequencing of Sporadic Amyotrophic Lateral Sclerosis Cerebrospinal Fluid Reveals Differentially Expressed miRNAs Related to Neural and Glial Activity. Front. Neurosci..

[24] Yelick J., Men Y., Jin S., Seo S., Espejo-Porras F., Yang Y. (2020). Elevated exosomal secretion of miR-124–3p from spinal neurons positively associates with disease severity in ALS. Exp. Neurol..

[25] Pinto S., Cunha C., Barbosa M., Vaz A.R., Brites D. (2017). Exosomes from NSC-34 Cells Transfected with hSOD1-G93A Are Enriched in miR-124 and Drive Alterations in Microglia Phenotype. Front. Neurosci..

[26] Vaz A.R., Cunha C., Gomes C., Schmucki N., Barbosa M., Brites D. (2015). Glycoursodeoxycholic acid reduces matrix metalloproteinase-9 and caspase-9 activation in a cellular model of superoxide dismutase-1 neurodegeneration. Mol. Neurobiol..

[27] Caldeira C., Oliveira A.F., Cunha C., Vaz A.R., Falcão A.S., Fernandes A., Brites D. (2014). Microglia change from a reactive to an age-like phenotype with the time in culture. Front. Cell Neurosci..

[28] Berthod F., Gros-Louis F., Maurer M.H. (2012). In Vivo and In Vitro Models to Study Amyotrophic Lateral Sclerosis. Amyotrophic Lateral Sclerosis (ALS).

[29] Hayden P.J., Harbell J.W. (2021). Special review series on 3D organotypic culture models: Introduction and historical perspective. Vitro Cell Dev. Biol. Anim..

[30] Yu J.Y., Chung K.H., Deo M., Thompson R.C., Turner D.L. (2008). MicroRNA miR-124 regulates neurite outgrowth during neuronal differentiation. Exp. Cell Res..

[31] Han D., Dong X., Zheng D., Nao J. (2019). MiR-124 and the Underlying Therapeutic Promise of Neurodegenerative Disorders. Front. Pharmacol..

[32] Kim K.Y., Kim Y.R., Choi K.W., Lee M., Lee S., Im W., Shin J.Y., Kim J.Y., Hong Y.H., Kim M. (2020). Downregulated miR-18b-5p triggers apoptosis by inhibition of calcium signaling and neuronal cell differentiation in transgenic SOD1 (G93A) mice and SOD1 (G17S and G86S) ALS patients. Transl. Neurodegener..

[33] Cho G.W., Kim G.Y., Baek S., Kim H., Kim T., Kim H.J., Kim S.H. (2011). Recombinant human erythropoietin reduces aggregation of mutant Cu/Zn-binding superoxide dismutase (SOD1) in NSC-34 cells. Neurosci. Lett..

[34] Hou Q., Ruan H., Gilbert J., Wang G., Ma Q., Yao W.D., Man H.Y. (2015). MicroRNA miR124 is required for the expression of homeostatic synaptic plasticity. Nat. Commun..

[35] Qin Z., Wang P.Y., Su D.F., Liu X. (2016). miRNA-124 in Immune System and Immune Disorders. Front. Immunol..

[36] Ge X.T., Lei P., Wang H.C., Zhang A.L., Han Z.L., Chen X., Li S.H., Jiang R.C., Kang C.S., Zhang J.N. (2014). miR-21 improves the neurological outcome after traumatic brain injury in rats. Sci. Rep..

[37] Munoz-Lasso D.C., Roma-Mateo C., Pallardo F.V., Gonzalez-Cabo P. (2020). Much More Than a Scaffold: Cytoskeletal Proteins in Neurological Disorders. Cells.

[38] Ruangjaroon T., Chokchaichamnankit D., Srisomsap C., Svasti J., Paricharttanakul N.M. (2017). Involvement of vimentin in neurite outgrowth damage induced by fipronil in SH-SY5Y cells. Biochem. Biophys. Res. Commun..

[39] Tilokani L., Nagashima S., Paupe V., Prudent J. (2018). Mitochondrial dynamics: Overview of molecular mechanisms. Essays Biochem..

[40] Liu W., Yamashita T., Tian F., Morimoto N., Ikeda Y., Deguchi K., Abe K. (2013). Mitochondrial fusion and fission proteins expression dynamically change in a murine model of amyotrophic lateral sclerosis. Curr. Neurovasc. Res..

[41] Westermann B. (2010). Mitochondrial fusion and fission in cell life and death. Nat. Rev. Mol. Cell Biol..

[42] Liu Y.J., McIntyre R.L., Janssens G.E., Houtkooper R.H. (2020). Mitochondrial fission and fusion: A dynamic role in aging and potential target for age-related disease. Mech. Ageing Dev..

[43] Barbosa M., Gomes C., Sequeira C., Goncalves-Ribeiro J., Pina C.C., Carvalho L.A., Moreira R., Vaz S.H., Vaz A.R., Brites D. (2021). Recovery of Depleted miR-146a in ALS Cortical Astrocytes Reverts Cell Aberrancies and Prevents Paracrine Pathogenicity on Microglia and Motor Neurons. Front. Cell Dev. Biol..

[44] Gurney M.E., Pu H., Chiu A.Y., Dal Canto M.C., Polchow C.Y., Alexander D.D., Caliendo J., Hentati A., Kwon Y.W., Deng H.X. (1994). Motor neuron degeneration in mice that express a human Cu,Zn superoxide dismutase mutation. Science.

[45] Turner B.J., Talbot K. (2008). Transgenics, toxicity and therapeutics in rodent models of mutant SOD1-mediated familial ALS. Prog. Neurobiol..

[46] Cavaliere F., Benito-Munoz M., Matute C. (2016). Organotypic Cultures as a Model to Study Adult Neurogenesis in CNS Disorders. Stem. Cells Int..

[47] Lossi L., Merighi A. (2018). The Use of ex Vivo Rodent Platforms in Neuroscience Translational Research With Attention to the 3Rs Philosophy. Front. Vet. Sci..

[48] Sofroniew M.V. (2020). Astrocyte Reactivity: Subtypes, States, and Functions in CNS Innate Immunity. Trends Immunol..

[49] Hopperton K.E., Mohammad D., Trepanier M.O., Giuliano V., Bazinet R.P. (2018). Markers of microglia in post-mortem brain samples from patients with Alzheimer’s disease: A systematic review. Mol. Psychiatry.

[50] Díaz-Amarilla P., Olivera-Bravo S., Trias E., Cragnolini A., Martinez-Palma L., Cassina P., Beckman J., Barbeito L. (2011). Phenotypically aberrant astrocytes that promote motoneuron damage in a model of inherited amyotrophic lateral sclerosis. Proc. Natl. Acad. Sci. USA.

[51] Staal J.A., Alexander S.R., Liu Y., Dickson T.D., Vickers J.C. (2011). Characterization of cortical neuronal and glial alterations during culture of organotypic whole brain slices from neonatal and mature mice. PLoS ONE.

[52] Gonzalez-Prieto M., Gutierrez I.L., Garcia-Bueno B., Caso J.R., Leza J.C., Ortega-Hernandez A., Gomez-Garre D., Madrigal J.L.M. (2021). MicroglialCX3CR1production increases in Alzheimer’s disease and is regulated by noradrenaline. Glia.

[53] Gomes C., Sequeira C., Barbosa M., Cunha C., Vaz A.R., Brites D. (2020). Astrocyte regional diversity in ALS includes distinct aberrant phenotypes with common and causal pathological processes. Exp. Cell Res..

[54] Lagos-Quintana M., Rauhut R., Yalcin A., Meyer J., Lendeckel W., Tuschl T. (2002). Identification of tissue-specific microRNAs from mouse. Curr. Biol..

[55] Krichevsky A.M., King K.S., Donahue C.P., Khrapko K., Kosik K.S. (2003). A microRNA array reveals extensive regulation of microRNAs during brain development. RNA.

[56] Ghafouri-Fard S., Shoorei H., Bahroudi Z., Abak A., Majidpoor J., Taheri M. (2021). An update on the role of miR-124 in the pathogenesis of human disorders. Biomed. Pharmacother..

[57] Zhao Y., Yan M., Chen C., Gong W., Yin Z., Li H., Fan J., Zhang X.A., Wang D.W., Zuo H. (2018). MiR-124 aggravates failing hearts by suppressing CD151-facilitated angiogenesis in heart. Oncotarget.

[58] Vuokila N., Lukasiuk K., Bot A.M., van Vliet E.A., Aronica E., Pitkanen A., Puhakka N. (2018). miR-124–3p is a chronic regulator of gene expression after brain injury. Cell Mol. Life Sci..

[59] An F.M., Gong G.H., Wang Y., Bian M., Yu L.J., Wei C.X. (2017). MiR-124 acts as a target for Alzheimer’s disease by regulating BACE1. Oncotarget.

[60] Bell E., Taylor M.A. (2017). Functional Roles for Exosomal MicroRNAs in the Tumour Microenvironment. Comput. Struct. Biotechnol. J..

[61] Diaz Quiroz J.F., Tsai E., Coyle M., Sehm T., Echeverri K. (2014). Precise control of miR-125b levels is required to create a regeneration-permissive environment after spinal cord injury: A cross-species comparison between salamander and rat. Dis. Model Mech..

[62] Edbauer D., Neilson J.R., Foster K.A., Wang C.F., Seeburg D.P., Batterton M.N., Tada T., Dolan B.M., Sharp P.A., Sheng M. (2010). Regulation of synaptic structure and function by FMRP-associated microRNAs miR-125b and miR-132. Neuron.

[63] Harrison E.B., Hochfelder C.G., Lamberty B.G., Meays B.M., Morsey B.M., Kelso M.L., Fox H.S., Yelamanchili S.V. (2016). Traumatic brain injury increases levels of miR-21 in extracellular vesicles: Implications for neuroinflammation. FEBS Open Bio.

[64] Fernandes A., Ribeiro A.R., Monteiro M., Garcia G., Vaz A.R., Brites D. (2018). Secretome from SH-SY5Y APP Swe cells trigger time-dependent CHME3 microglia activation phenotypes, ultimately leading to miR-21 exosome shuttling. Biochimie.

[65] Strickland I.T., Richards L., Holmes F.E., Wynick D., Uney J.B., Wong L.F. (2011). Axotomy-induced miR-21 promotes axon growth in adult dorsal root ganglion neurons. PLoS ONE.

[66] Xue Q., Yu C., Wang Y., Liu L., Zhang K., Fang C., Liu F., Bian G., Song B., Yang A. (2016). miR-9 and miR-124 synergistically affect regulation of dendritic branching via the AKT/GSK3beta pathway by targeting Rap2a. Sci. Rep..

[67] Osking Z., Ayers J.I., Hildebrandt R., Skruber K., Brown H., Ryu D., Eukovich A.R., Golde T.E., Borchelt D.R., Read T.A. (2019). ALS-Linked SOD1 Mutants Enhance Neurite Outgrowth and Branching in Adult Motor Neurons. iScience.

[68] Wang G., Huang Y., Wang L.-L., Zhang Y.-F., Xu J., Zhou Y., Lourenco G.F., Zhang B., Wang Y., Ren R.-J. (2016). MicroRNA-146a suppresses ROCK1 allowing hyperphosphorylation of tau in Alzheimer’s disease. Sci. Rep..

[69] Gatto R.G., Amin M.Y., Deyoung D., Hey M., Mareci T.H., Magin R.L. (2018). Ultra-High Field Diffusion MRI Reveals Early Axonal Pathology in Spinal Cord of ALS mice. Transl. Neurodegener..

[70] Zayia L.C., Tadi P. (2021). Neuroanatomy, Motor Neuron. StatPearls [Internet].

[71] Nagata K., Hama I., Kiryu-Seo S., Kiyama H. (2014). microRNA-124 is down regulated in nerve-injured motor neurons and it potentially targets mRNAs for KLF6 and STAT3. Neuroscience.

[72] Hawley Z.C.E., Campos-Melo D., Droppelmann C.A., Strong M.J. (2017). MotomiRs: miRNAs in Motor Neuron Function and Disease. Front. Mol. Neurosci..

[73] Genç B., Jara J.H., Lagrimas A.K., Pytel P., Roos R.P., Mesulam M.M., Geula C., Bigio E.H., Ozdinler P.H. (2017). Apical dendrite degeneration, a novel cellular pathology for Betz cells in ALS. Sci. Rep..

[74] De Vos K.J., Hafezparast M. (2017). Neurobiology of axonal transport defects in motor neuron diseases: Opportunities for translational research?. Neurobiol. Dis..

[75] Hardiman O., Al-Chalabi A., Chio A., Corr E.M., Logroscino G., Robberecht W., Shaw P.J., Simmons Z., van den Berg L.H. (2017). Amyotrophic lateral sclerosis. Nat. Rev. Dis. Primers.

[76] Warita H., Itoyama Y., Abe K. (1999). Selective impairment of fast anterograde axonal transport in the peripheral nerves of asymptomatic transgenic mice with a G93A mutant SOD1 gene. Brain Res..

[77] De Vos K.J., Chapman A.L., Tennant M.E., Manser C., Tudor E.L., Lau K.F., Brownlees J., Ackerley S., Shaw P.J., McLoughlin D.M. (2007). Familial amyotrophic lateral sclerosis-linked SOD1 mutants perturb fast axonal transport to reduce axonal mitochondria content. Hum. Mol. Genet..

[78] Delic V., Kurien C., Cruz J., Zivkovic S., Barretta J., Thomson A., Hennessey D., Joseph J., Ehrhart J., Willing A.E. (2018). Discrete mitochondrial aberrations in the spinal cord of sporadic ALS patients. J. Neurosci. Res..

[79] Onesto E., Colombrita C., Gumina V., Borghi M.O., Dusi S., Doretti A., Fagiolari G., Invernizzi F., Moggio M., Tiranti V. (2016). Gene-specific mitochondria dysfunctions in human TARDBP and C9ORF72 fibroblasts. Acta Neuropathol. Commun..

[80] Magrane J., Cortez C., Gan W.B., Manfredi G. (2014). Abnormal mitochondrial transport and morphology are common pathological denominators in SOD1 and TDP43 ALS mouse models. Hum. Mol. Genet..

[81] Joshi A.U., Saw N.L., Vogel H., Cunnigham A.D., Shamloo M., Mochly-Rosen D. (2018). Inhibition of Drp1/Fis1 interaction slows progression of amyotrophic lateral sclerosis. EMBO Mol. Med..

[82] Liu K., Yan L., Jiang X., Yu Y., Liu H., Gu T., Shi E. (2017). Acquired inhibition of microRNA-124 protects against spinal cord ischemia-reperfusion injury partially through a mitophagy-dependent pathway. J. Thorac. Cardiovasc. Surg..

[83] Veremeyko T., Kuznetsova I.S., Dukhinova M., Yung A.W.Y., Kopeikina E., Barteneva N.S., Ponomarev E.D. (2019). Neuronal extracellular microRNAs miR-124 and miR-9 mediate cell-cell communication between neurons and microglia. J. Neurosci. Res..

[84] Zullo J., Matsumoto K., Xavier S., Ratliff B., Goligorsky M.S. (2015). The cell secretome, a mediator of cell-to-cell communication. Prostaglandins Lipid Mediat..

[85] Meyer K., Kaspar B.K. (2017). Glia-neuron interactions in neurological diseases: Testing non-cell autonomy in a dish. Brain Res..

[86] Vaz A.R., Pinto S., Ezequiel C., Cunha C., Carvalho L.A., Moreira R., Brites D. (2019). Phenotypic Effects of Wild-Type and Mutant SOD1 Expression in N9 Murine Microglia at Steady State, Inflammatory and Immunomodulatory Conditions. Front. Cell Neurosci..

[87] Gugliandolo A., Giacoppo S., Bramanti P., Mazzon E. (2018). NLRP3 Inflammasome Activation in a Transgenic Amyotrophic Lateral Sclerosis Model. Inflammation.

[88] Franklin T.C., Wohleb E.S., Zhang Y., Fogaca M., Hare B., Duman R.S. (2018). Persistent Increase in Microglial RAGE Contributes to Chronic Stress-Induced Priming of Depressive-like Behavior. Biol. Psychiatry..

[89] Guzmán-Lenis M.S., Navarro X., Casas C. (2009). Drug screening of neuroprotective agents on an organotypic-based model of spinal cord excitotoxic damage. Restor. Neurol. Neurosci..

[90] Zhang J., Liu Y., Liu X., Li S., Cheng C., Chen S., Le W. (2018). Dynamic changes of CX3CL1/CX3CR1 axis during microglial activation and motor neuron loss in the spinal cord of ALS mouse model. Transl. Neurodegener..

[91] Slota J.A., Booth S.A. (2019). MicroRNAs in Neuroinflammation: Implications in Disease Pathogenesis, Biomarker Discovery and Therapeutic Applications. Noncoding RNA.

[92] Saura J., Tusell J.M., Serratosa J. (2003). High-yield isolation of murine microglia by mild trypsinization. Glia.

[93] Lossi L., Alasia S., Salio C., Merighi A. (2009). Cell death and proliferation in acute slices and organotypic cultures of mammalian CNS. Prog. Neurobiol..

[94] Vaz A.R., Falcão A.S., Scarpa E., Semproni C., Brites D. (2020). Microglia Susceptibility to Free Bilirubin Is Age-Dependent. Front. Pharmacol..

[95] Popko J., Fernandes A., Brites D., Lanier L.M. (2009). Automated analysis of NeuronJ tracing data. Cytometry A.

[96] Silva S.L., Vaz A.R., Diógenes M.J., van Rooijen N., Sebastião A.M., Fernandes A., Silva R.F., Brites D. (2012). Neuritic growth impairment and cell death by unconjugated bilirubin is mediated by NO and glutamate, modulated by microglia, and prevented by glycoursodeoxycholic acid and interleukin-10. Neuropharmacology.

