# Supplementary material for: Overexpression of miR-124 in Motor Neurons Plays a Key Role in ALS Pathological Processes
Source: Int J Mol Sci. 2021 Jun 7;22(11):6128. doi: 10.3390/ijms22116128 (PMC8201298; doi:10.3390/ijms22116128)
Supplement: Supplementary file 1 [file ijms-22-06128-s001.zip › ijms-1242427-supplementary.pdf]

International Journal of
Molecular Sciences

Article – Supplementary information

Overexpression of miR-124 in Motor Neurons Plays a Key Role in ALS Pathological Processes

Supplementary Figures

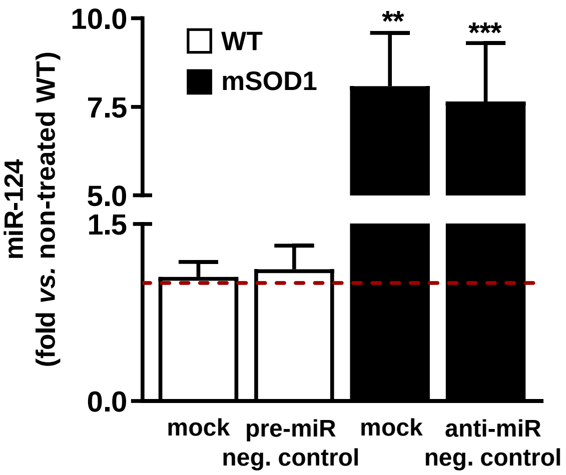

Figure S1. MiR-124 expression is not affected by the transfection method either in WT and mSOD1 MNs. White bars represent WT MNs treated with XtremeGENE™ HP DNA Transfection Reagent (mock control) or modulated with Pre-miR™ Negative Control. Black bars represent mSOD1 MNs treated with XtremeGENE™ HP DNA Transfection Reagent (mock control) or modulated with Anti-miR™ Negative Control. Results are expressed as fold change relatively to WT MNs incubated with Optimem (dashed red line). **p<0.01 vs. WT MNs. WT, wild type; MN, motor neurons (NSC-34 cell line); mSOD1, MNs overexpressing G93A mutation in super-oxide dismutase 1.

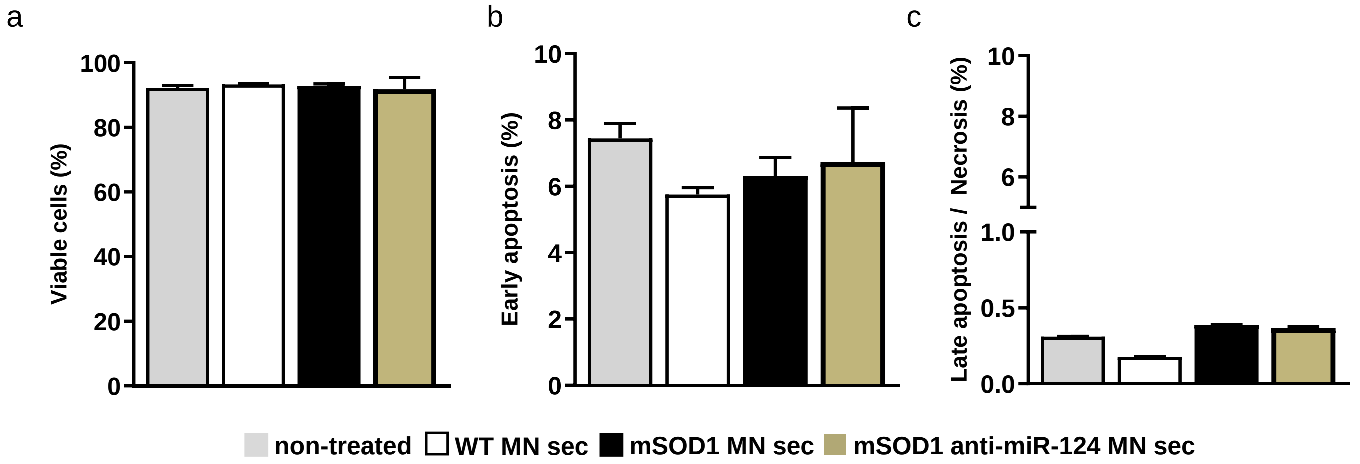

Figure S2. Microglia viability is not affected by the presence of neuronal secretome, derived from either WT, mSOD1 or anti-miR-124 mSOD1 MNs. Two days in vitro primary spinal microglia were incubated with WT MN, mSOD1 MN secretome or with the secretome from anti-miR-124 mSOD1 MNs for 4 h. Non-treated cells were used as controls. After incubation, the percentage of viable, early apoptotic, and late-apoptotic/necrotic cells were determined by flow cytometry with phycoerythrin-conjugated annexin V (annexinV-PE) and 7-amino-actinomycin D (7-AAD). The three populations were distinguished as follows: (**a**) viable cells (annexin V-PE and 7-AAD negative), (**b**) early apoptotic cells (annexinV-PE positive and 7-AAD negative), and (**c**) cells in late stages of apoptosis or necrosis (annexinV-PE and 7-AAD positive). Results are mean± SEM from at least 3 independent experiments performed in duplicate. WT, wild type; MN, motor neurons (NSC-34 cell line); mSOD1, MNs overexpressing G93A mutation in superoxide dismutase 1.

Supplementary Tables

Table S1. List of primer sequences used in RT-qPCR to amplify miRNA.

miRNA	Target sequence (5′-3′)
hsa-miR-124-3p	UAAGGCACGCGGUGAAUGCC
hsa -miR-125b-5p	UCCCUGAGACCCUAACUUGUGA
hsa miR-146a-5p	UGAGAACUGAAUUCCAUGGGUU
hsa miR-21-5p	UAGCUUAUCAGACUGAUGUUGA
SNORD110	Reference gene
RNU1A1	Reference gene

RT-qPCR, real-time quantitative polymerase chain reaction; miRNA, microRNA; hsa, homo sapiens.

Table S2. List of primer sequences used in RT-qPCR to amplify protein-coding genes.

Gene	Forward primer (5′-3′)	Reverse primer (5′-3′)
Synaptophysin	GACGTTGGTAGTGCCTGTGA	GCACAGGAAAGTAGGGGGTC
DLG4	GAGGCTGGCGGCCAGTACACCAG	ACAGAGCAGGCGGTCAG
Kif5b	GGTCCTACAGTTGCCACCTA	ATTGAAATACGCCAGGCCCA
Dynein	GCCTCAGTCTCTGTCCCATC	AAGTCCTGGGGTAAGGTGCT
IL-1β	CAGGCTCCGAGATGAACAAC	GGTGGAGAGCTTTCAGCTCATA
IL-18	TGGTTCCATGCTTTCTGGACTCCT	TTCCTGGCCAAGAGGAAGTG
HMGB1	CTCAGAGAGGTGGAAGACCATGT	GGGATGTAGGTTTTCATTTCTCTTTC
iNOS	ACCCACATCTGGCAGAATGAG	AGCCATGACCTTTCGCATTAG
IL-10	ATGCTGCTTGCTCTTACTGA	GCAGCTCTAGGAGCATGTGG
CX3CL1	CTCACGAATCCCAGTGGCTT	TTTCTCCTTCGGGTCAGCAC
S100B	GAGAGAGGGTGACAAGCACAA	GGCCATAAACTCCTGGAAGTC
GFAP	CAAACTGGCTGATGTCTACC	GCTTCATCTGCCTCCTGTCTA
CX3CR1	ATGGGGTCTCTGTCTGCTCT	TACTGGCAATGGGTGGCATT
MFG-E8	AGCCTGAATGGTAGGGTTGG	GAGACTGCATCCTGCAACCA
β-actin	GCTCCGGCATGTGCAA	AGGATCTTCATGAGGTAGT

DLG4, discs large MAGUK scaffold protein 4 (that encodes for postsynaptic density protein 95, PSD-95); Kif5b encodes for the protein kinesin -1 heavy chain; IL, interleukin; HMGB1, high mobility group box 1; iNOS, inducible nitric oxide synthase; MFG-E8, milk fat globule-EGF factor 8 protein, CX3CL1, C-X3-C motif chemokine ligand 1/fractalkine; S100B, S100 calcium-binding protein B; GFAP, glial fibrillary acidic protein; CX3CR1, C-X3-C motif chemokine receptor 1; β-actin, beta-actin.

Table S3. List of antibodies used for immunocytochemistry (ICC) or immunohistochemistry (IHC).

	Antibodies	Source	Species	Dilution	Assay
Primary	Anti-β-III tubulin	Merck Millipore, MAB1637	Mouse	1:500	ICC
	Anti- mitofusin 2	AbCam, ab50838	Rabbit	1:50	ICC
	Anti-Drp1	AbCam, ab140494	Mouse	1:150	ICC
	Anti-iNOS	BDBiosciences, 610329	Mouse	1:100	ICC
	Anti-Arg 1	Santa Cruz, sc18355	Goat	1:50	ICC
	Anti-GFAP	NovoCastra, GFAP-GA5-6035278	Mouse	1:100	IHC
	Anti-Iba1	Wako, 019-19741	Rabbit	1:250	IHC
Secondary	AlexaFluor 488 anti-mouse	Invitrogen, A-10680	Goat	1:1000	IHC
	AlexaFluor 488 anti-rabbit	Invitrogen, A-11008	Goat	1:1000	IHC
	AlexaFluor 594 anti-mouse	Invitrogen, A-11005	Goat	1:1000	IHC
	AlexaFluor 594 anti-goat	Invitrogen, A-21468	Chicken	1:1000	IHC
	AlexaFluor 647 anti-mouse	Invitrogen, A-21236	Goat	1:500	IHC
	AlexaFluor 594 anti-rabbit	Invitrogen, A-11012	Goat	1:500	IHC

Arg1, arginase 1; Drp 1, dynamin-related protein 1; GFAP, glial fibrillary acidic protein; iNOS, inducible nitric oxide synthase; Iba-1, ionized calcium-binding adapter molecule 1.

Table S4. Absolute values of the heatmap indicated in Figure 6g for mSDO1 and mSDO1+anti-miR-124 MN secretome versus WT spinal cord slices.

	**mSOD1 (fold change *vs.* WT)** Mean ± SEM	**mSOD1 + anti-miR-124 MN secretome (fold change *vs. WT*)** Mean ± SEM
**Genes**		
iNOS	**3.71 ± 1.32**	**1.26 ± 0.41**
IL-1β	**2.99 ± 0.44**	**0.70 ± 0.18**
IL-10	**2.72 ± 0.42**	**0.51 ± 0.08**
HMGB1	**0.54 ± 0.11**	**0.82 ± 0.07**
CX3CL1	**0.71 ± 0.07**	0.85 ± 0.08
PSD-95	**0.42 ± 0.06**	**0.81 ± 0.10**
Synaptophysin	**1.61 ± 0.18**	**0.83 ± 0.12**
S100B	1.09 ± 0.06	**0.84 ± 0.05**
GFAP	**0.42 ± 0.08**	**1.28 ± 0.28**
MFG-E8	0.75 ± 0.07	0.79 ± 0.33
CX3CR1	**1.94 ± 0.36**	**0.83 ± 0.09**
**Inflamma-miRs**		
hsa-miR-124-3p	**1.57 ± 0.14**	**0.74 ± 0.02**
hsa-miR-125b-5p	0.94 ± 0.13	0.95 ± 0.21
hsa-miR-146a-5p	**1.85 ± 0.09**	1.75 ± 0.25
hsa-miR-21-5p	**2.16 ± 0.40**	2.38 ± 0.39
PI^+ cells	**1.65 ± 0.14**	**0.81 ± 0.13**

Organotypic cultures were obtained from the spinal cord of 10–12-weeks-old mice, as described in methods. Slices were collected for necrosis evaluation and mRNA expression of iNOS, IL-1β, IL-10, HMGB1, CX3CL1, PSD-95, Synaptophysin, S100B, GFAP, MFG-E8, and CX3CR1 by reverse transcriptase quantitative real-time PCR (RT-qPCR), using β-actin as reference gene. Results are expressed as fold change versus WT organotypic slices incubated with MN culture media (control) and are mean ± SEM. The values considered statically significant are in bold. iNOS, inducible nitric oxide synthase; IL-1β, interleukin-1 beta; IL-10, interleukin-10; HMGB1, high mobility group protein 1; CX3CL1, C-X3-C motif chemokine ligand 1; PSD-95, postsynaptic density protein 95 (gene Dlg4); S100B, S100 calcium-binding protein B; GFAP, glial fibrillary acidic protein; MFG-E8, milk fat globule-EGF factor 8; CX3CR1, C-X3-C motif chemokine receptor 1; mSOD1, MNs overexpressing G93A mutation in superoxide dismutase 1; PI, propidium iodide.
